# A Modified Novel Validated High-Throughput Hemagglutinin Inhibition Assay Using Recombinant Virus-like Particles and Human Red Blood Cells for the Objective Evaluation of Recombinant Hemagglutinin Nanoparticle Seasonal Influenza Vaccine

**DOI:** 10.3390/microorganisms12112358

**Published:** 2024-11-19

**Authors:** Timothy S. Vincent, Mingzhu Zhu, Anand Parekh, Urvashi Patel, Shane Cloney-Clark, Andrew Klindworth, David Silva, Andrew Gorinson, Karlee Miranda, Mi Wang, Zachary Longacre, Bin Zhou, Iksung Cho, Rongman Cai, Raj Kalkeri, Louis Fries, Vivek Shinde, Joyce S. Plested

**Affiliations:** 1 Clinical Immunology, Novavax, Gaithersburg, MD 20878, USA; mzhu@novavax.com (M.Z.); aparekh@novavax.com (A.P.); upatel@novavax.com (U.P.); wcloneyclark@novavax.com (S.C.-C.); aklindworth@novavax.com (A.K.); dsilva@novavax.com (D.S.); agorinson@novavax.com (A.G.); kmiranda@novavax.com (K.M.); mwang@novavax.com (M.W.); rkalkeri@novavax.com (R.K.); 2 Vaccine Development, Novavax, Gaithersburg, MD 20878, USA; zlongacre@novavax.com; 3 Preclinical Development, Vaccine Immunology, Novavax, Gaithersburg, MD 20878, USA; bzhou@novavax.com; 4 Biostatistics, Novavax, Gaithersburg, MD 20878, USA; icho@novavax.com (I.C.); rcai@novavax.com (R.C.); 5 Contractor, Novavax, Gaithersburg, MD 20878, USA; lfries@novavax.com; 6 Clinical Development, Novavax, Gaithersburg, MD 20878, USA; vshinde@novavax.com

**Keywords:** hemagglutination inhibition, immunogenicity, influenza vaccine, influenza virus, singleton, validation, virus-like particles, human red blood cells, egg-derived influenza viruses, CypherOne™ Hemagglutination Analyzer (InDevR, Boulder, CO, USA)

## Abstract

Currently available seasonal influenza vaccines confer variable protection due to antigenic changes resulting from the accumulation of diverse mutations. The analysis of new seasonal influenza vaccines is challenging in part due to the limitations of the traditional hemagglutination inhibition (HAI) assay with A/H3N2 strains. An improved and objective novel HAI assay was developed with recombinant virus-like particles (VLPs) and an egg-derived virus as agglutinins, the oseltamivir treatment of VLPs, human red blood cells, and using an automated image reader-based analysis of hemagglutination. HAI validation was demonstrated using four VLPs and egg-derived strains, with 46–56 serum samples tested 12 times in duplicate per strain. The validated HAI assay was precise as indicated by the percent geometric coefficient of variation for intra-, inter-, and total assay precision, as well as accurate as evidenced by percent bias measurements. The assay exhibited linearity, specificity for homologous type/subtype strains, and sensitivity with a starting dilution of 1:10. Assay robustness and sample stability were demonstrated as a percentage difference compared to reference condition. Validated HAI results were equivalent for the single and duplicate sample testing and correlated well with a qualified live wild-type influenza microneutralization assay. These findings demonstrate the suitability of this high-throughput novel modified validated HAI assay for evaluating vaccine immunogenicity and efficacy.

## 1. Introduction

Seasonal influenza virus infections pose a significant threat to public health globally, resulting in 3–5 million cases of severe illness and 290,000–650,000 respiratory deaths annually [1]. Annual vaccination is recommended as the most effective approach for the prevention and control of seasonal influenza [1,2]. Annually updated seasonal influenza vaccines confer variable protection due to the continual genetic changes in circulating viruses, underscoring the challenges in controlling influenza infections and the associated morbidity [3,4,5].

The viral hemagglutinin (HA) surface glycoproteins are key determinants of vaccine efficacy against seasonal circulating strains of influenza [6]. The hemagglutination inhibition (HAI) assay represents the gold standard method for determining an influenza vaccine-elicited immune response and quantitative antibody titers for the influenza virus [7,8,9].

New influenza vaccine development is challenging due, in part, to traditional, labor-intensive, and cumbersome HAI assay methods involving avian red blood cells (RBCs), complex sample preparation, subjectivity, and low assay throughput. In addition, certain clades of A/H3N2 viruses agglutinate avian RBCs poorly and alternative assays such as virus neutralization assays must be used [10,11,12]. A modified novel HAI assay protocol has been developed to generate more reliable and consistent data using human RBCs [13], avoiding partial hemagglutination or irregular shapes of RBC precipitation with avian-origin RBCs [14]. A novel automated CypherOne™ Hemagglutination Analyzer (InDevR, Boulder, CO, USA) has also been used to determine and provide a visual permanent record of assay results to eliminate the need for tilting of the HAI plate before image interpretation, thereby simplifying the assay and reducing assay turnaround time [14]. A recent report employed a combination of guinea pig RBCs (instead of avian RBCs), neuraminidase (NA) inhibitor oseltamivir (to prevent NA-mediated agglutination), and CypherOne™ (InDevR, Boulder, CO, USA) HAI plate reader-based automation but focused solely on the HAI assay for H3N2 influenza strains [15]. Although all these studies have reported reproducible and reliable results, there is a need to develop a robust high-throughput HAI assay method for both the H1N1 and H3N2 strains as well as B-type viruses by harmonizing the existing protocols.

This study describes an enhanced novel HAI assay method developed using human RBCs (Type O) and egg-derived influenza viruses or Baculovirus/Sf9-produced recombinant virus-like particles (VLPs) for agglutination, the oseltamivir treatment of the virus/VLPs, and CypherOne™ Hemagglutination Analyzer (InDevR, Boulder, CO, USA) to determine the titers of HAI antibodies in human serum. Procedurally, the egg-derived virus HAI and the VLP HAI are identical except for the choice of agglutinin. The use of VLPs as agglutinins in the HAI assay allows for wild-type virus sequences to be utilized while avoiding potential serological bias associated with the egg-adapted mutations of the egg-derived viruses. In addition, as VLPs are non-infectious, they can be used as pandemic strains thus avoiding the need for higher biosafety level containment. Traditionally, in HAI assays, sample titration is performed in duplicates (variability within 2-fold is considered acceptable) [16,17], which is time-consuming and escalates the assay cost when handling large quantities of clinical samples. In a clinical study, qNIV-E-301 (NCT04120194), only 12 (0.0276%) samples or controls out of the 21,728 samples and controls tested in duplicates for the four homologous (seasonal) strains had variability exceeding 2-fold between duplicates. The extremely low proportion of samples with more than 2-fold variability between the two replicates suggested the possibility of reliably testing samples in singleton with comparable precision and accuracy to those of testing in duplicates.

The present study describes the validation of the HAI assay using egg-derived influenza viruses or recombinant VLPs as agglutinins and human RBCs (hemagglutination indicator particle) for the measurement of anti-influenza HA antibody titers in human serum, with the primary aim of demonstrating its suitability for testing sera from influenza vaccine clinical trials. The study also evaluated the testing of human serum samples in singleton compared to duplicates in the influenza VLP HAI assay. The titers from the validated HAI assay correlated well with those of a qualified microneutralization (MN) assay.

## 2. Materials and Methods

### 2.1. Seasonal Influenza Viruses and VLPs

The validation of HAI assay for egg-derived influenza viruses was performed using the following four vaccine-homologous 2019–2020 Northern Hemisphere seasonal strains: A/Kansas/14/2017 (A/H3N2), A/Brisbane/02/2018 (A/H1N1), B/Maryland/15/2016 (B/Victoria Lineage), and B/Phuket/3073/2013 (B/Yamagata Lineage). VLPs were produced in Sf9 insect cells infected with baculoviruses bearing cloned genes of influenza HA, neuraminidase (NA), and M1 [18,19]. The membrane-bound VLPs were approximately 100 nM in diameter and morphologically resembled influenza virus particles; the NA was enzymatically active [18,20]. The VLP HAI assay validation included four recombinant Baculovirus/Sf9–produced VLPs [20] corresponding to the aforementioned homologous seasonal influenza vaccine strains and four VLPs corresponding to the following four antigenically drifted (heterologous) A(H3N2) viruses: A/California/94/2019, A/Cardiff/0508/2019, A/Netherlands/1268/2019, and A/Tokyo/EH1801/2018.

### 2.2. Human RBCs

Human RBCs (Type O) with K_3_EDTA as an anticoagulant (Biological Specialty Corporation/BioIVT, Westbury, NY, USA, cat. HUMANRBK3-0101896) were used for the HAI assay. Each new donor’s erythrocytes were screened for comparability using a panel of previously tested sera, at least 80% of which must return titers within 2-fold of the prior values for acceptance of the new donor. Each washed and diluted lot of 0.75% red blood cell suspension is tested with negative and positive quality control sera to confirm consistent behavior before use in the assay.

### 2.3. Test Serum Samples

The serum samples used in the assay validation experiments were from healthy humans (BioIVT, Westbury, NY, USA) self-reporting receipt of the influenza vaccine within 1 year before sample collection, human sera that showed positive HAI during the pre-validation serum screening processes, and HA-specific sheep serum samples from virus HA-hyperimmune sheep obtained from the National Institute for Biological Standards and Control and Novavax influenza vaccine non-clinical studies. Negative serum was HA antibody-depleted/stripped human serum (Valley Biomedical, Winchester, VA, USA, cat. HS1200W) diluted 1:2 in PBS. The depletion of HA antibodies from human sera was performed using a negative selection by HA–cross-linked agarose beads (by Novavax, Inc., Gaithersburg, MD, USA). All serum samples used in the assay validation are listed in Table S1.

### 2.4. HAI Assay Procedure

The HAI assay was conducted per the WHO Manual for the laboratory diagnosis and virology surveillance of influenza [21], with some modifications (such as human RBCs and the automated assay readout method mentioned below). To remove nonspecific inhibitors of HA, the serum samples were incubated at 37 °C for 18 to 20 h with a receptor-destroying enzyme (RDE; Denka Seiken Co., Ltd., San Jose, CA, USA, cat. 370013) diluted 1:4 in Dulbecco’s Phosphate Buffered Saline with calcium and magnesium (DPBS; Quality Biological, Gaithersburg, MD, USA, cat. 114-059-101), followed by heat-inactivation at 56 °C for 30 ± 2 min and further diluted to 1:10 with DPBS. In the HAI assay, each RDE-treated sample was diluted 1:2 serially in a 96-well U-bottom Microtiter™ plate (Thermo Fisher Scientific, Waltham, MA, USA, cat. 2205) for a total of 10 dilutions with DPBS. The viral stock was adjusted to 4 hemagglutination (HAg) units in DPBS with 80 nM of oseltamivir to prevent possible NA-mediated agglutination (NA inhibitor; ChemScene, Monmouth Junction, NJ, USA, cat. CS-0553). After incubating with 4 HAg units of the virus, the sample–virus mixture was then incubated with 0.75% human RBCs (BioIVT, cat. HUMANRBK3-0101896) for 80 to 100 min at room temperature (RT). To determine serum antibody titers, the HAI assay plates were scored using the automated and validated CypherOne™ Hemagglutination Analyzer (InDevR Inc., Boulder, CO, USA, software versions 3.2.0.0 and 4.0.0.19). The HAI titers were determined from the reciprocal of the highest serum sample dilution that completely prevented hemagglutination.

### 2.5. Validation Parameters: Precision, Specificity, Linearity, Sensitivity, Assay Robustness, and Sample Stability

The assay parameters evaluated for each vaccine-homologous strain of egg-derived virus and VLP agglutinins were precision, specificity, linearity, sensitivity (lower limit of quantitation [LLOQ]), robustness, and sample stability. The intra-, inter-, and total-assay precision were tested at both the individual sample and strain levels, representing the overall assay variance of all samples tested for each egg-derived virus/VLP strain. Strain-specific sheep sera were used to test the ability of the HAI assay to measure and differentiate the influenza virus type- and subtype-specific antibodies (anti-A/H1N1 [A strain subtype 1 HA], anti-A/H3N2 [A strain subtype 3 HA], and anti-B strain HA antibodies). Negative human serum samples included in the specificity runs were expected to consistently test negative (geometric mean titer [GMT] of 5–7). To determine the assay linearity, for each strain, two influenza HAI-positive samples were tested undiluted and further serially diluted in 1:2 dilution series (from 1:2 up to 1:256) in negative serum, with a target of a minimum of six dilutions above the LLOQ. Samples were tested in replicates in a total of six runs by two analysts over 3 days. The expected HAI titers at each dilution were calculated from the overall HAI GMT from all runs of the undiluted sample divided by the dilution factor. The accuracy of the linearity at each dilution was evaluated in terms of percent relative bias, where values of 100% or −50% correspond to a titer that is 2-fold higher or 2-fold lower, respectively, than the expected titer.

The assay sensitivity was determined in terms of the LLOQ, which is the lowest HAI titer with acceptable precision and accuracy. The LLOQ was determined for samples assayed in the linearity tests. Precision (percent geometric coefficient of variation [%GCV] of HAI titers) and accuracy (percent relative bias) were estimated across the runs for these samples.

Robustness was tested for all egg-derived virus/VLP strains to evaluate the effect of RBC age (storage time from receipt/collection time), serum–agglutinin incubation time, and serum–agglutinin–RBC incubation time (plate reading time) on the HAI results. For the RBC suspension storage time robustness assay, the panel of serum samples were tested using 0.75% RBC stored at 2 to 8 °C for 2 weeks (14 ± 3 days) and 0.75% RBC prepared from 10% RBC suspension stored at 2 to 8 °C for 2 weeks (14 ± 3 days). The HAI GMT of the test storage conditions was compared with the overall HAI GMT from the precision assay runs (utilizing fresh 0.75% RBCs stored at 2 to 8 °C for less than a week [≤7 days]; baseline).

Plate reading time robustness was determined at 75, 90, 120, and 150 min after the addition of the 0.75% RBC suspension to the serum–virus mixture (incubated for 60 min). The HAI GMTs were compared with the standard 90-min reading time. To evaluate the combined serum–agglutinin incubation time and plate reading time robustness, serum–agglutinin incubation time was varied (50, 60, and 70 min) and, for each duration of incubation, the plate reading time was varied (75, 90, 120, and 150 min) after the addition of RBCs. Baseline HAI GMTs were from assay runs in which serum–agglutinin incubation time was 60 min and the plate reading time was 90 min after RBC addition. Additionally, for egg-derived virus/VLP titration and back-titration (virus/VLP titration plate reading time robustness), the plate reading time was determined at 90 ± 10 min following RBC addition.

The stability of RDE-treated samples stored at 2 to 8 °C (1 and 2 months), ≤−20 °C (2 months), and subjected to two freeze/thaw cycles was assessed and compared with the overall HAI GMT from precision runs (baseline; with samples stored at 2 to 8 °C for ≤7 days). The stability of neat (undiluted) serum samples stored at −20 ± 10 °C (1 month) and subjected to seven freeze/thaw cycles (from storage in a −80 ± 10 °C freezer) was assessed using three quality control (QC) samples for each egg-derived virus/VLP strain. The results were compared with the overall GMT from the precision runs (baseline) as described above.

### 2.6. Singleton (Single Titer) Testing of Serum Samples in the Influenza VLP HAI Assay

To analyze the influence of singleton testing on individual samples (single titer, i.e., singlicate), the validation data of the VLP HAI assay for the four homologous seasonal vaccine strains were used and reproduced. The GMT and %GCV were determined for the validation dataset of each vaccine-homologous strain. For each serum sample, the GMT and %GCV were determined for (1) singleton titers (N = 24), (2) paired replicate (duplicate) GMT (N = 12), and (3) random titer replicates (duplicate) GMT (N = 12). Random titer pairings were intended to simulate potential titer pairings based on the data collected in the validation studies.

The percentage difference in GMT for randomly chosen replicates (random replicate 1 and random replicate 2) was determined relative to the GMT of paired replicates as shown below.

(1) $$\%\;\mathrm{Difference}=100\;\times \;\frac{\left(\mathrm{Random}\;\mathrm{HAI}\;\mathrm{GMT}-\mathrm{Paired}\;\mathrm{HAI}\;\mathrm{GMT}\right)}{\mathrm{Paired}\;\mathrm{HAI}\;\mathrm{GMT}}$$

For assessing the influence of singleton results and paired duplicates on precision (%GCV), 36 serum samples were tested for the four vaccine-homologous strains. The % difference for %GCV of singleton titers and random replicates was calculated relative to the %GCV of paired replicates as shown below.

(2) $$\%\;\mathrm{Difference}=100\;\times \;\frac{\left(\mathrm{Paired}\;\mathrm{or}\;\mathrm{Random}\;\%\mathrm{GCV}-\mathrm{Singleton}\;\%\mathrm{GCV}\right)}{\mathrm{Singleton}\;\%\mathrm{GCV}}$$

For evaluating the influence of singleton testing on clinical study results, HAI antibody titer data corresponding to VLPs from the Phase 3 clinical study qNIV-E-301 [22] were utilized. Aside from the aforementioned four vaccine-homologous seasonal influenza strains and the four heterologous viruses, this clinical study included three additional heterologous wild-type strains (A/South Australia/34/2019, A/Idaho/13/2018, and B/Washington/02/2019). Antibody titers using VLP HAI obtained in duplicate (n = 2) from approximately 1286 test subjects and two study protocol arms (2019–2020 Fluzone^®^ Quadrivalent and Quad-NIV) were used to model the influence of reporting single titer (n = 1) results (randomly selected from one of the paired duplicates) instead of the GMT of paired duplicate (n = 2) values. The per-protocol population included 1279 to 1286 test subjects (two time points: Day 0 [pre-vaccination] and Day 28 [post-vaccination]; two vaccination groups: 2019–2020 Fluzone Quadrivalent and Quad-NIV). The GMT (by strain and vaccine group) was calculated as the reported GMT (duplicate) values of the antibody titers using wild-type sequence VLP-HAI. The results of GMT calculated using paired replicates (duplicates) were compared with the GMT calculated using individual titers that were randomly selected. The randomly selected titer GMT results were determined twice (Random titers 1 and 2). The geometric mean ratio (GMR; post-/pre-vaccination) were determined using the reported GMT from paired duplicate samples [22] and randomly selected single titers. The random titer pairings included were intended to simulate potential titer pairings based on the data collected in clinical studies.

The percent seroprotection rate (%SPR) was determined using data from the study qNIV-E-301. The percentage of results with titer ≥1:40 was determined using the reported GMT from paired duplicate samples and using randomly selected single titers (Random 1 and Random 2) for subject visits on Day 28. The percent seroconversion rate (%SCR) was also determined using qNIV-E-301 study data. The results (%) for subject visits on Day 0 (unvaccinated) and Day 28 (vaccinated) where the titer or GMT on Day 28 was higher by ≥4-fold were determined using the reported GMT from paired duplicate samples and using randomly selected single titers (Random 1 and Random 2).

### 2.7. Correlation Analysis of the HAI Assay and MN Assay

Serum samples were assessed in the HAI assay (using the same methodology as for validation) and in a qualified influenza MN assay for each indicated wild-type strain (A/Brisbane/02/2018, A/Kansas/14/2017, and B/Maryland/15/2016). The results from the final titers of both assays were compared to determine the correlation between them.

### 2.8. Statistical Analysis

Statistical analyses of the validation results were performed using SAS^®^ version 9.4 (SAS Institute Inc., Cary, NC, USA) in a Windows (Microsoft Corp., Redmond, WA, USA) environment. The intra- and inter-assay precision were assessed by determining the %GCV through the variance component analysis with the sample as a fixed effect and analyst and day as random effects. The correlation between the HAI and MN assay results was determined by performing linear regression analysis using GraphPad Prism^®^ software version 9.3.1 (GraphPad Software, San Diego, CA, USA).

## 3. Results

### 3.1. Assay Validation Parameters

#### 3.1.1. Precision

For the egg-derived HAI assay, all four seasonal influenza homologous virus strains met the acceptance criteria of intra-, inter-, and total-assay precision ≤ 50% GCV for at least 80% of the samples tested for each strain (A/Kansas/14/2017: 98.2% of the samples, A/Brisbane/02/2018: 95.7%, B/Maryland/15/2016: 97.8%, B/Phuket/3073/2013: 100%) (Table 1). Similarly, for the VLP HAI assays, intra-, inter-, and total-assay precision were ≤50% GCV for 100% of the samples for A/Kansas/14/2017, A/Brisbane/02/2018, A/Netherlands/1268/2019, and A/Tokyo/EH1801/2018 strains; and >93% of the samples for B/Maryland/15/2016, B/Phuket/3073/2013, A/California/94/2019, and A/Cardiff/0508/2019 strains. Additionally, all egg-derived virus/VLP strains met the acceptance criteria of ≤30% GCV for the overall intra-, inter-, and total-assay precision (Table 2).

#### 3.1.2. Specificity

In the specificity analysis, the overall HAI GMT from the strain-homologous immune serum was ≥4-fold higher than from the heterologous serum, thereby meeting the acceptance criterion for both egg-derived and VLP HAI assays. For the egg-derived assay, the overall HAI GMT of subtype/lineage homologous serum was at least 8-fold higher than that of the heterologous serum (A/Kansas/14/2017: 8-fold, A/Brisbane/02/2018: 300-fold, B/Maryland/15/2016: 500-fold, B/Phuket/3073/2013: 20-fold; Table 3). For the VLP HAI assay, the overall HAI GMT of the subtype/lineage homologous serum was ≥4-fold higher than the heterologous serum HAI GMT (A/Kansas/14/2017: 13-fold, A/Brisbane/02/2018: 180-fold, B/Maryland/15/2016: 95-fold, B/Phuket/3073/2013: 33-fold, A/California/94/2019: 22-fold, A/Cardiff/0508/2019: 17-fold, A/Netherlands/1268/2019: 9-fold, A/Tokyo/EH1801/2018: 18-fold; Table 4). The heterologous sera were either HAI-negative (i.e., HAI GMT of 5) or had low HAI titers compared to the homologous sera. Negative control human serum samples (depleted of anti-HA antibodies) consistently tested negative (GMT of 5).

#### 3.1.3. Linearity and LLOQ

**Figure 1:**
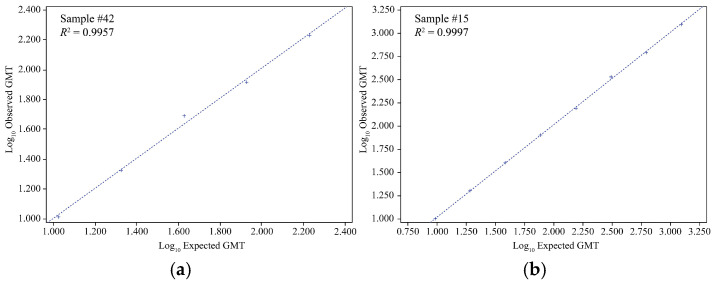

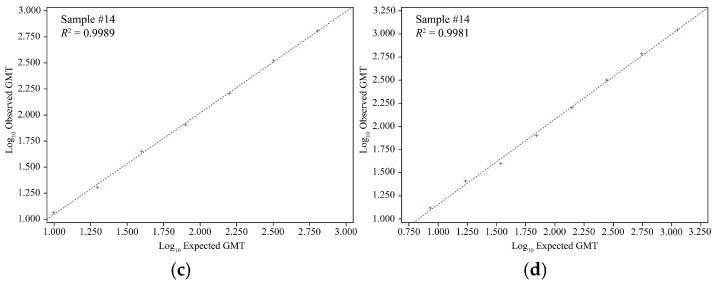
Linearity results of egg-derived virus HAI assay for influenza virus strains. (**a**) A/Kansas/14/2017, (**b**) A/Brisbane/02/2018, (**c**) B/Maryland/15/2016, and (**d**) B/Phuket/3073/2013. GMT, geometric mean titer; HAI, hemagglutination inhibition; *R*^2^, coefficient of determination.

**Figure 2:**
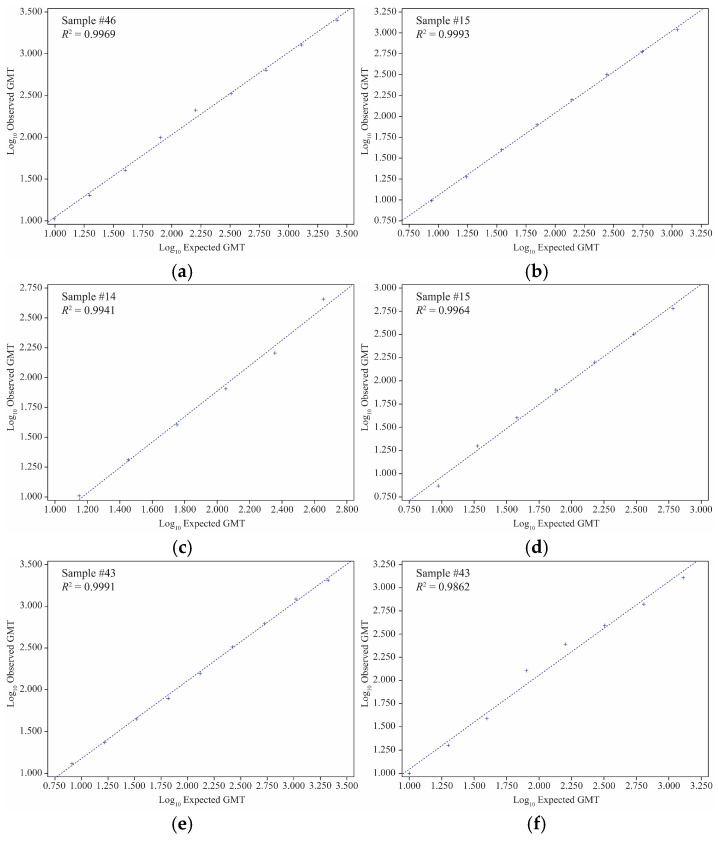

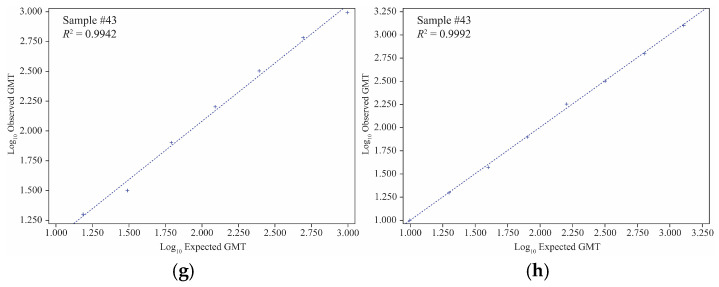
Linearity results of VLP HAI assay for strains. (**a**) A/Kansas/14/2017, (**b**) A/Brisbane/02/2018, (**c**) B/Maryland/15/2016, (**d**) B/Phuket/3073/2013, (**e**) A/California/94/2019, (**f**) A/Cardiff/0508/2019, (**g**) A/Netherlands/1268/2019, and (**h**) A/Tokyo/EH1801/2018. GMT, geometric mean titer; HAI, hemagglutination inhibition; *R*^2^, coefficient of determination; VLP, virus-like particle.

All the virus strain samples with an expected HAI GMT of ≥8 were included in the linear regression and percent relative bias analyses. The linearity of both the egg-derived HAI and VLP HAI assays was successfully demonstrated, with *R*^2^ ranging from 0.9814 to 0.9999 and from 0.9777 to 0.9999, respectively, for all individual samples examined for each egg-derived virus or VLP agglutinin, meeting the acceptance criterion (regression line *R*^2^ ≥ 0.95) (Figure 1 and Figure 2; Tables S2 and S3). Also, the percent relative bias for all samples (for both the egg-derived virus and VLP agglutinins) was in the acceptance range (−50% to 100% of the expected HAI GMT). In the egg-derived HAI, the %GCV of all samples was <50%. In the VLP HAI, the %GCV was <50% for all the sample dilutions, except for one sample dilution for B/Phuket/3073/2013 VLP (%GCV of 54.5) (acceptance criteria: %GCV ≤ −60% (Tables S4 and S5).

All samples were diluted to an HAI titer of 10, the lowest titer (GMT) that met the acceptance criteria of intra-, inter-, and total-assay %GCV ≤ 60% and percent relative bias of between −50 and 100. Hence, LLOQ was set at 10 for the egg-derived virus/VLP HAI assay.

#### 3.1.4. Robustness

Robustness analysis of the RBC suspension storage time (2 weeks) for the egg-derived virus HAI showed that all samples (100%) tested for all four virus strains exhibited an HAI GMT % difference ranging from −50% to 100% (2-fold difference) compared to the baseline. These findings suggested that RBC suspension can be stored at 2 to 8 °C for up to 2 weeks before use (acceptance criteria: at least 80% of samples with HAI GMT within the 2-fold difference of baseline). In the VLP HAI assay, the majority of the samples (except three) tested for all four VLP strains had a HAI GMT % difference within the range of −50% to 100% compared to the baseline. However, three of the four strains showed an asymmetric tendency to yield a predominance of −50% to 0% change, suggesting the suitability of RBC suspension storage conditions of 2 to 8 °C for a maximum of 1 week (7 days) before use (Table S6).

Robustness analysis of the plate reading time (75-, 120-, and 150-min) demonstrated an HAI GMT % difference within the range of −50% to 100% compared to the 90 min plate reading reference, suggesting that, in both the egg-derived virus/VLP HAI assays, the plates can be read within a range of 75 min and 150 min after RBC addition (Table S7). In the combined robustness analysis of serum–virus/VLP incubation time and plate reading time, the HAI GMT % difference of all sample HAI titers read at 75, 90, 120, and 150 min after 50 min to 70 min of egg-derived virus/VLP plus serum incubation were within 2-fold (−50% to 100%) of the baseline reading (Table S8). However, HAI titers showed more variations when longer incubation times were combined (such as 70 min of egg-derived virus/VLP–serum incubation and 150 min of egg-derived virus/VLP–serum–RBC incubation [plate reading time]), although the HAI GMT % difference was still within 2-fold of the baseline. These findings suggest that experimental conditions with egg-derived virus/VLP–serum incubation time between 50 and 60 min, as well as a plate reading time of 75 to 120 min, can be considered as best practice to maintain result consistency in HAI assays (Table S8). Furthermore, the robustness of the plate reading time was also evaluated for 90 ± 10 min after RBC incubation for virus titration and back-titration. Consistent HAI titers were observed at 80 and 100 min post-RBC addition (in line with those at standard 90 min), suggesting that both the egg-derived virus and VLP HAI assay titration plates can be read at 90 ± 10 min of RBC incubation.

#### 3.1.5. Stability

In the RDE-treated sample stability analysis, HAI GMTs of at least 80% of the samples were within the 2-fold difference of the baseline (−50% to 100%) following storage at 2 to 8 °C for 1 month and 2 months, storage at ≤−20 °C for 2 months, and after undergoing two freeze/thaw cycles in both HAI assays (Table S9). However, in the egg-derived virus HAI assay, the HAI GMT of samples with 2 months of storage showed increasing variability relative to the baseline and a trend towards decreasing titers compared to 1 month of storage, especially for the A/Kansas/14/2017 and B/Phuket/3073/2013 assays. These findings suggest that to maintain consistency for both egg-derived virus and VLP HAI assays, the storage of RDE-treated samples at 2 to 8 °C should be limited to 1 month prior to testing (Table S9).

Stability analysis of the neat (undiluted) serum samples was included to assess sample stability under the potential storage conditions commonly observed in clinical trials. The HAI GMTs of all samples were within the range of −50% to 100% difference of the baseline following seven freeze/thaw cycles and 1 month of storage in a −20 °C freezer, suggesting that the serum samples remained suitable for both the egg-derived virus/VLP HAI assay testing even after undergoing these conditions.

### 3.2. Influence of Singleton (Single Titer) Sample Testing on GMT of Serum Samples

The GMT of the singleton titers and paired replicates were identical for all samples with all four homologous seasonal influenza strains. Also, there was no significant difference in the GMT of randomly combined sample pairs compared to that of the singleton or paired titers. The randomly chosen replicates showed a %GMT difference of −2.85% to 5.95%, −2.85% to 9.05%, −5.61% to 2.93%, and −5.61% to 5.95% compared to that of the paired replicates for A/Kansas/14/2017, A/Brisbane/02/2018, B/Phuket/3073/2013, and B/Maryland/15/2016, respectively. These differences in GMT were minimal and not significant (within the acceptable 2-fold assay variability). Thus, the random pairing of titer results does not significantly impact the GMT of either the paired or individual titers when assessed at the sample level.

### 3.3. Influence of Singleton (Single Titer) Results and Paired Duplicates (Mean of Two Titers) on Precision (%GCV) at the Serum Sample Level

Inspection of the individual sample level results for all four vaccine-homologous strains (A/Kansas/14/2017, A/Brisbane/02/2018, B/Phuket/3073/2013, and B/Maryland/15/2016) suggests that the use of singleton titers, paired replicates, or random replicate data has little influence on precision, as can be seen in Tables S10–S13. Overall precision for each vaccine-homologous strain was virtually unaffected by the various replicate strategies as assessed by %GCV at the inter-, intra-, and total-assay levels (Table 5).

### 3.4. Influence of Singleton (Single Titer) Testing on Clinical Study GMT Results

There was no noticeable difference in the GMT determined using the paired duplicate results or randomly selected titers for all the VLP strains and vaccine groups (Table 6; such as for A/Kansas/14/2017: GMT [95% confidence interval {CI}] at Day 28 for 2019–2020 Fluzone Quadrivalent vaccine groups were 90.7 [84.9, 96.9], 91.3 [85.5, 97.6], and 90.8 [85.0, 96.9], respectively, for duplicates, random 1 and random 2). Since the GMTs were very close (no statistical difference) with an almost overlapping 95% CI between the singleton and duplicates, testing samples in singleton had no appreciable influence on clinical GMT results.

### 3.5. Influence of Singleton (Single Titer) Testing on Clinical Study GMR, SPR and SCR Results

**Figure 3:**
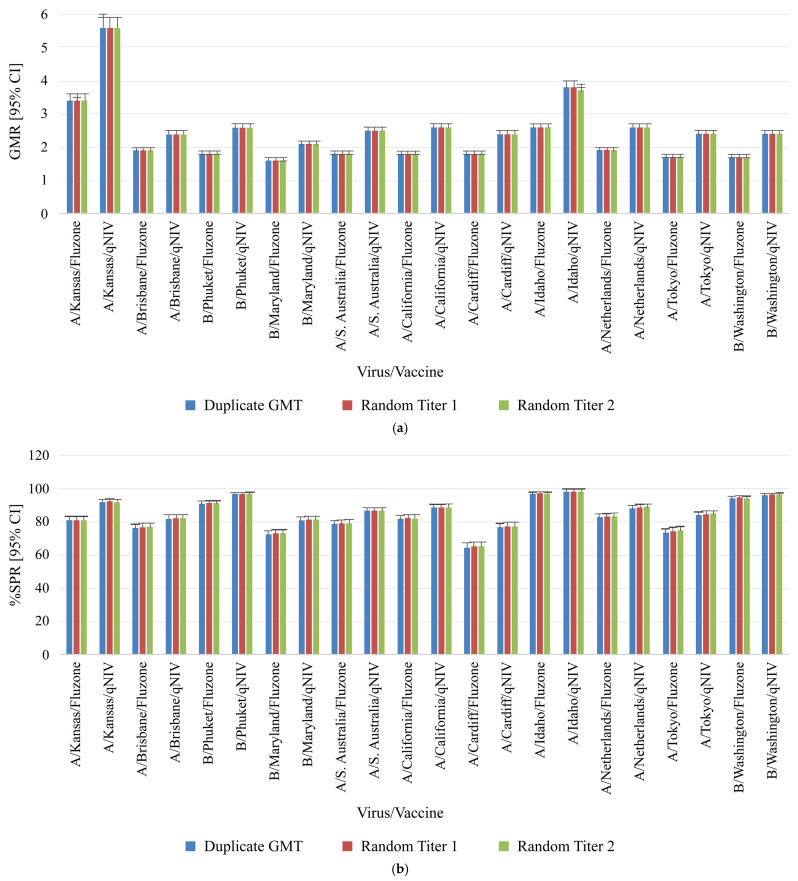

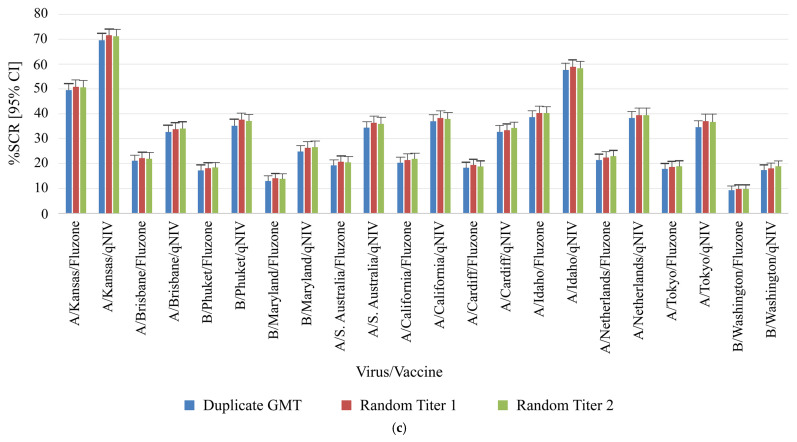
Comparison of clinical samples from qNIV-E-301 using duplicate results compared to random titer (singleton) for key study parameters. (**a**) Geometric mean ratio, (**b**) Seroprotection rate, and (**c**) Seroconversion rate for homologous and drifted seasonal influenza strains. Geometric mean ratio (GMR) was defined as the ratio of post-vaccination and pre-vaccination HAI GMTs within the same treatment group [23]. Seroprotection was defined as a titer of ≥1:40 (a titer that gives a 50% reduction of disease) [7]. Seroconversion was defined as HAI titer post-vaccination meeting one of the following criteria: either pre-vaccination titer < 1:10 and post-vaccination titer ≥ 1:40, or pre-vaccination titer ≥ 1:10 and at least a 4-fold increase in post-vaccination titer [24,25]. CI, confidence interval; GMR, geometric mean ratio; GMT, geometric mean titer; HAI, hemagglutination inhibition; SCR, seroconversion rate; SPR, seroprotection rate; VLP, virus-like particle.

The GMR, %SPR, as well as %SCR, and their corresponding 95% CI from paired duplicates or randomly selected single titers were very similar for each virus strain and protocol arm pair (Figure 3). Overall, these findings showed that reporting titers in singleton had no impact on the four key clinical metrics of GMT, GMR, SPR, and SCR.

### 3.6. Correlation Between VLP-Based HAI and Wild-Type Virus MN Assays

**Figure 4:**
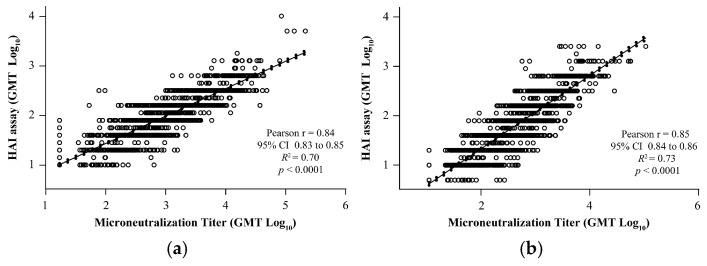

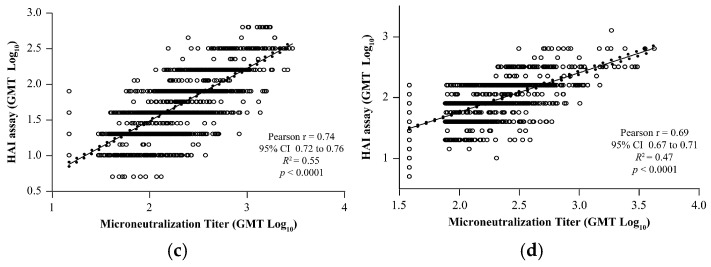
Correlation analysis of the validated HAI assay with qualified MN assay for strains. (**a**) A/Brisbane/02/2018, (**b**) A/Kansas/14/2017, (**c**) B/Maryland/15/2016, and (**d**) B/Phuket/3073/2013. The dotted line shows 95% CI. CI, confidence interval; GMT, geometric mean titer; HAI, hemagglutination inhibition; MN, microneutralization; *R*^2^, coefficient of determination.

To evaluate the concordance of the HAI results with the MN assay, correlation analysis was performed using four different influenza strains. Results of the HAI for both A and B strains demonstrated a significant positive correlation with the qualified MN assays: A/Brisbane/02/2018 (Pearson’s *r* = 0.84, *R*^2^ = 0.70, *p* < 0.0001; Figure 4a), A/Kansas/14/2017 (Pearson’s *r* = 0.85, *R*^2^ = 0.73, *p* < 0.0001; Figure 4b), B/Maryland/15/2016 (Pearson’s *r* = 0.74, *R*^2^ = 0.55, *p* < 0.0001; Figure 4c), and B/Phuket/3073/2013 (Pearson’s *r* = 0.69, *R*^2^ = 0.47, *p* < 0.0001; Figure 4d).

## 4. Discussion

The data presented in the current work describes the development and validation of a novel robust HAI assay using egg-derived viruses/recombinant VLPs with human RBCs to assess the immunogenicity of seasonal influenza vaccines in clinical trial settings. To our knowledge, this is the first report that uniquely demonstrates the validation of HAI using human RBCs and an automated HAI reader suitable for clinical samples. The HAI assay showed acceptable precision, specificity, linearity, sensitivity, and robustness (RBC age [storage time], serum–virus/VLP incubation time, plate reading time) for the egg-derived viruses as well as the recombinant VLP strains. Furthermore, the singleton analyses using data from the VLP HAI validation did not significantly impact the GMT or precision (%GCV) at the individual sample level. Similarly, using the HAI titer data for wild-type sequence VLP from the Phase 3 clinical study qNIV-E-301 [22] showed no adverse impact of reporting titers in singleton on all four key clinical metrics (GMT, GMR, SPR, and SCR). We have demonstrated that our novel modification of HAI is strongly predictive of influenza neutralizing activity in sera (Figure 4) and have also explored the relationship between HAI titers using A(H3N2) VLPs and egg-derived viruses, which showed a strong positive correlation as well as a regression slope close to 1 (see Figure S1 in Supplementary Materials). We also note that the original historic driver of the adoption of HAI assays lay in their ability to predict neutralization data and protection (at the in vitro and animal challenge levels. We believe that the data presented in Figure 4 of the present study strongly validate the HAI assay as fulfilling this function. We compared a Cypher One–based readout and the manual reading of the assays using human RBCs during assay development (Table S14) and demonstrated equivalency through bridging experiments.

The source and the quality of the RBCs as critical reagents are reported as critical factors in the HAI assay [26]. In general, RBCs of avian or animal origin are used in HAI assays [15,16,27]. The selection of the appropriate species source of RBCs for the HAI assay remains a challenge given the failure of some recent H3N2 influenza viruses to agglutinate the RBC of common reagent species and non-specific inhibition of hemagglutination (because of non-specific serum interference with hemagglutination of avian RBCs) [13,14,28]. To circumvent these challenges, the present assay protocol used human RBCs (Type O) for the measurement of HAI antibody titers in human serum. Human influenza viruses preferentially bind to α2,6-linked sialic acid (SA) molecules [11,29]. Human RBCs (Type O) and guinea pig RBCs have more α2,6-linked SA molecules on their surfaces compared with that of avian RBCs [11,30]. Makkoch et al. showed that human RBCs (Type O) that lack preexisting antibodies against the pandemic influenza H1N1 virus yielded HAI titers most comparable to those obtained with turkey RBCs [31].

Additionally, the assay employed the NA inhibitor oseltamivir to prevent NA-mediated agglutination, which further strengthened the antigenic characterization of HA proteins of seasonal influenza viruses. Although the HAI GMT % difference (−50% to 100%) for both the egg-derived and VLP HAI assay results leveraged the use of RBC suspensions stored at 2 to 8 °C for up to 2 weeks, an asymmetric change/trend (−50% to 0%) in HAI GMT was noted for three strains in the VLP HAI assay. These findings recommend a maximum shelf-life of 1 week (7 days) as the most conservative condition for RBC suspension storage (2 to 8 °C) in the HAI assays. Our study results are in line with a previous study that reported a shelf-life of up to 1 week for human erythrocytes [13]. Additionally, it might be useful to prescreen RBCs from donors and virus combinations using QC samples before testing clinical trial sera to avoid any non-specific virus–RBC interaction.

The lack of harmonized and standardized assay readouts can introduce a high degree of variability in HAI antibody titer interpretation. The tilting of HAI plates at a 45 ± 60° angle to check for a “teardrop pattern” in case of non-specific inhibition with avian RBCs is a commonly followed practice in many research laboratories [14]. Similar to Wilson et al. [14], the present study used an automated platform CypherOne^TM^ Hemagglutination Analyzer for reading HAI plates. The benefits of this platform are numerous, including the following: increased objectivity; standardization, consistency of titer interpretation; improved data integrity; reduced assay time, testing costs, possible data transcription errors; and the elimination of subjective biases in the manual scoring of plates as well as a permanent visual image/record to refer back to [14,15]. Together, these factors indicate that the CypherOne^TM^ platform adds significant value to clinical sample testing.

Validation of the HAI method ensures the reliability and reproducibility of results [13,15,16,32]. Our study validated the egg-derived virus/recombinant VLP HAI assay for precision, specificity, linearity, sensitivity (LLOQ), RBC storage time, serum–virus/VLP incubation time, plate reading time, and serum sample storage and freeze/thaw effects on serum samples. A comprehensive understanding of these validation parameters is crucial for the assessment of clinical samples and assay reliability. In our study, the two HAI methods using human RBCs and either egg-derived influenza viruses or recombinant VLPs met the precision and accuracy criteria in measuring antibody titers in human serum, with overall assay %GCV being <30% for all egg-derived virus/VLP strains, and % relative bias within the range of −50% to 100% (2-fold). Neat (undiluted) sample stability findings showed that serum samples could be used in the HAI assays after up to seven freeze/thaw cycles and after 1 month of storage in a −20 °C freezer. These observations indicate that the validated HAI assays can be used in clinical trials to assess the immunogenicity of egg-derived and recombinant influenza vaccines.

Furthermore, testing samples in singleton in HAI assays can offer advantages in terms of reduced clinical testing time and costs, increased sample throughput, lower resource needs, and improved time management. Similarly, utilizing the HAI titer data of wild-type sequence VLP HAI from the Phase 3 clinical study and reporting titers from singleton showed no influence on the four key clinical analysis metrics such as GMT, GMR, SPR, and SCR. This suggests that the seasonal influenza VLP HAI assay samples (pre- and post-vaccination) can be tested routinely in singleton, and the final results can be reported as titers without adversely affecting the data readout and interpretation.

The HAI assay is a more commonly used serological technique for assessing influenza-specific humoral immunity than the MN assay [33]. The HAI assay identifies antibodies that bind to the globular head of viral HA and prevent virus-mediated RBC agglutination, while the MN assay detects functional neutralizing antibodies inhibiting the virus entry/replication in mammalian cells [34], which may have a broader range of specificities. Given these differences, establishing correlations between the HAI and MN assays would be valuable for comparing vaccine assessments and gaining a comprehensive understanding of immune responses to influenza vaccines [34]. Our study results revealed a significant correlation between the validated HAI assay and a qualified MN assay (for both A and B strains). These findings indicate that the validated HAI assay results align with other measures of the immune response against seasonal influenza. Strong correlation observed between HAI and MN results in our studies are consistent with previous publications (Veguilla et al. [35] and Trombetta et al. [34]), which also showed strong positive correlations between the HAI and MN assays for both the A and B influenza strains.

## 5. Conclusions

In conclusion, the validated egg-derived virus and VLP HAI assays with human RBCs performed similarly with comparable precision and accuracy, indicating the suitability for evaluating humoral immune response (HAI) for seasonal influenza in human serum samples from clinical studies. Furthermore, there was no difference in the precision, GMR, SPR, and SCR with singleton determination of titer testing compared with duplicate titer HAI testing. These findings suggest that testing in singleton can offer cost-effectiveness and improve assay throughput, without impacting key study parameters and conclusions from clinical data analysis.

## Figures and Tables

**Table 1:** Summary of results of egg-derived virus and VLP HAI assay sample inter-assay, intra-assay, and total precision for homologous and drifted seasonal influenza strains.

Virus Strains	HAI Assay Type	Total Sample (N)	Proportion of Samples Within Acceptance Criteria, n (%)		
			Intra-Assay %GCV ≤ −50%	Inter-Assay %GCV ≤ −50%	Total %GCV ≤ −50%
A/Kansas/14/2017	Egg-derived	56	56 (100.0)	56 (100.0)	55 (98.2)
	VLP	46	46 (100.0)	46 (100.0)	46 (100.0)
A/Brisbane/02/2018	Egg-derived	46	46 (100.0)	44 (95.7)	44 (95.7)
	VLP	46	46 (100.0)	46 (100.0)	46 (100.0)
B/Maryland/15/2016	Egg-derived	46	46 (100.0)	46 (100.0)	45 (97.8)
	VLP	46	46 (100.0)	45 (97.8)	44 (95.7)
B/Phuket/3073/2013	Egg-derived	46	46 (100.0)	46 (100.0)	46 (100.0)
	VLP	46	46 (100.0)	45 (97.8)	45 (97.8)
A/California/94/2019	VLP	48	48 (100.0)	47 (97.9)	45 (93.8)
A/Cardiff/0508/2019		48	48 (100.0)	47 (97.9)	47 (97.9)
A/Netherlands/1268/2019		48	48 (100.0)	48 (100.0)	48 (100.0)
A/Tokyo/EH1801/2018		48	48 (100.0)	48 (100.0)	48 (100.0)

When the HAI titer was <10, a value of 5 was used for calculation purposes. %GCV, percent geometric coefficient of variation; HAI, hemagglutination inhibition; VLP, virus-like particle.

**Table 2:** Summary of results of egg-derived virus and VLP HAI assay overall precision for homologous and drifted seasonal influenza strains.

Virus Strains	HAI Assay Type	Intra-Assay %GCV	Inter-Assay %GCV	Total %GCV
A/Kansas/14/2017	Egg-derived	16.2	3.9	16.7
	VLP	17.0	5.1	17.8
A/Brisbane/02/2018	Egg-derived	16.2	2.2	16.3
	VLP	14.1	3.4	14.6
B/Maryland/15/2016	Egg-derived	12.4	3.0	12.8
	VLP	16.4	4.8	17.1
B/Phuket/3073/2013	Egg-derived	16.0	7.1	17.6
	VLP	11.2	3.3	11.7
A/California/94/2019	VLP	18.8	10.4	21.6
A/Cardiff/0508/2019		19.7	12.2	23.3
A/Netherlands/1268/2019		13.2	3.9	13.8
A/Tokyo/EH1801/2018		15.0	4.6	15.7

In the egg-derived virus HAI assay, overall assay precision was evaluated by testing 46 serum samples for each virus strain, except for A/Kansas/14/2017 virus for which 56 samples were tested. In the VLP HAI assay, precision was evaluated by testing 46 serum samples for each homologous VLP strain, and 48 samples for antigenically drifted VLPs. When the HAI titer was <10, a value of 5 was used for calculation purposes. %GCV, percent geometric coefficient of variation; HAI, hemagglutination inhibition; VLP, virus-like particle.

**Table 3:** Summary of the results of HAI assay subtype/lineage-level specificity for seasonal influenza egg-derived viruses using human RBCs for H3N2, H1N1, B/Victoria, and B/Yamagata influenza strains.

Sample	HA of Virus Type	Overall HAI GMT			
		A/Kansas/14/2017 (H3N2)	A/Brisbane/02/2018 (H1N1)	B/Maryland/15/2016 (B/Victoria)	B/Phuket/3073/2013 (B/Yamagata)
Sample #47	H3N2	1280	5	6	5
Sample #29	H3N2	2080	5	5	5
Sample #27	H3N2	1280	5	5	5
Sample #43	H3N2	452	5	5	5
Sample #49	H1N1	160	1567	7	7
Sample #28	H1N1	5	2070	5	5
Sample #45	B/Victoria	160	5	2560	310
Sample #32	B/Victoria	46	5	2944	368
Sample #41	B/Yamagata	160	5	5	18,784
Sample #31	B/Victoria	80	5	1280	904

Geometric mean titer (GMT) was defined as the antilog of the mean of the log-transformed HAI titers for a given treatment group [23]. HAI titer of <10 was defined as 5 for calculation purposes. The bold numbers indicate the HAI results from homologous virus and antiserum pair. GMT, geometric mean titer; HA, hemagglutinin; HAI, hemagglutination inhibition; RBCs, red blood cells.

**Table 4:** Summary of results of HAI assay subtype/lineage-level specificity for seasonal influenza VLP using human RBCs for H3N2, H1N1, B/Victoria, and B/Yamagata influenza strains.

Sample	HA of Virus Type	Overall HAI GMT							
		A/Kansas/14/2017 (H3N2)	A/Brisbane/02/2018 (H1N1)	B/Maryland/15/2016 (B/Victoria)	B/Phuket/3073/2013 (B/Yamagata)	A/California/94/2019 (H3N2)	A/Cardiff/0508/2019 (H3N2)	A/Netherlands/1268/2019 (H3N2)	A/Tokyo/EH1801/2018 (H3N2)
Sample #47	H3N2	2032	5	26	92	359	302	311	185
Sample #29	H3N2	3116	5	5	5	1514	1429	206	5
Sample #27	H3N2	1318	5	5	5	1318	508	854	381
Sample #43	H3N2	1437	5	5	5	8366	1280	3948	2560
Sample #49	H1N1	147	2560	15	80	127	76	78	39
Sample #28	H1N1	5	2850	5	5	5	5	5	5
Sample #45	B/Victoria	101	14	2487	190	120	78	80	40
Sample #32	B/Victoria	5	5	4663	309	5	5	5	5
Sample #41	B/Yamagata	78	5	5	10,240	170	71	127	78
Sample #31	B/Victoria	5	5	960	55	5	5	5	5
Sample #51	H3N2	–	–	–	–	2487	640	–	1437
Sample #50	H3N2	–	–	–	–	3836	–	–	622
Sample #52	H3N2	–	–	–	–	–	359	1208	–
Sample #53	H3N2	–	–	–	–	–	1356	640	–

Geometric mean titer (GMT) was defined as the antilog of the mean of the log-transformed HAI titers for a given treatment group [23]. HAI titer of <10 was defined as 5 for calculation purposes. The bold numbers indicate the HAI results from homologous virus and antiserum pair. GMT, geometric mean titer; HA, hemagglutinin; HAI, hemagglutination inhibition; RBC, red blood cells; VLP, virus-like particle.

**Table 5:** Overall results for precision (%GCV) for singleton VLP HAI titers and replicate (duplicate) GMT for four homologous influenza strains.

Strain	Parameter	%GCV		
		Intra-Assay	Inter-Assay	Total-Assay
A/Kansas/14/2017	Singleton titers	16.0	5.5	16.9
	Paired replicates	16.0	5.5	16.9
	Random replicates 1	16.6	5.5	17.5
	Random replicates 2	16.5	5.2	17.3
A/Brisbane/02/2018	Singleton titers	15.8	3.0	16.1
	Paired replicates	15.8	3.0	16.1
	Random replicates 1	16.2	4.2	16.8
	Random replicates 2	16.2	2.8	16.4
B/Maryland/15/2016	Singleton titers	17.1	6.8	18.4
	Paired replicates	17.1	6.8	18.4
	Random replicates 1	16.9	6.5	18.2
	Random replicates 2	17.0	6.7	18.3
B/Phuket/3073/2013	Singleton titers	10.1	2.9	10.6
	Paired replicates	10.1	2.9	10.6
	Random replicates 1	10.5	2.9	10.9
	Random replicates 2	10.5	2.9	10.9

%GCV, percent geometric coefficient of variation; GMT, geometric mean titer; HAI, hemagglutination inhibition; VLP, virus-like particle.

**Table 6:** Comparison of qNIV-E-301 GMT of HAI antibody titers for duplicate and randomly selected titers (singleton) from VLP HAI clinical testing for selected homologous and drifted seasonal influenza strains.

Strain	Protocol Arm (N)	Visit	GMT (95% CI)		
			Duplicate	Random Titer 1	Random Titer 2
A/Kansas/14/2017	Fluzone Quadrivalent (1286)	Day 0	26.5 (25.4, 27.7)	26.5 (25.3, 27.7)	26.5 (25.4, 27.7)
		Day 28	90.7 (84.9, 96.9)	91.3 (85.5, 97.6)	90.8 (85.0, 96.9)
	Quad-NIV (1280)	Day 0	27.3 (26.1, 28.6)	27.4 (26.1, 28.7)	27.4 (26.1, 28.7)
		Day 28	153.6 (143.9, 163.9)	153.2 (143.6, 163.5)	153.5 (143.8, 163.8)
A/Brisbane/02/2018	Fluzone Quadrivalent (1286)	Day 0	32.4 (30.7, 34.2)	32.4 (30.7, 34.2)	32.3 (30.6, 34.1)
		Day 28	62.7 (59.2, 66.4)	62.8 (59.3, 66.5)	62.5 (59.0, 66.2)
	Quad-NIV (1280)	Day 0	31.7 (30.0, 33.5)	31.7 (30.0, 33.4)	31.7 (30.1, 33.5)
		Day 28	76.6 (72.4, 81.1)	76.4 (72.2, 80.9)	76.5 (72.3, 81.0)
B/Maryland/15/2016	Fluzone Quadrivalent (1286)	Day 0	29.5 (28.3, 30.8)	29.6 (28.3, 30.9)	29.5 (28.3, 30.8)
		Day 28	47.2 (45.2, 49.4)	47.4 (45.3, 49.6)	47.5 (45.4, 49.6)
	Quad-NIV (1280)	Day 0	29.8 (28.5, 31.1)	29.8 (28.5, 31.2)	29.7 (28.4, 31.1)
		Day 28	62.8 (59.8, 66.0)	62.6 (59.7, 65.8)	63.0 (60.0, 66.1)
B/Phuket/3073/2013	Fluzone Quadrivalent (1286)	Day 0	44.3 (42.7, 46.1)	44.3 (42.6, 46.0)	44.2 (42.5, 46.0)
		Day 28	78.4 (75.1, 81.9)	78.3 (75.0, 81.8)	78.8 (75.4, 82.3)
	Quad-NIV (1280)	Day 0	45.8 (44.0, 47.7)	45.8 (44.0, 47.7)	46.0 (44.1, 47.9)
		Day 28	118.3 (113.0, 123.8)	118.0 (112.8, 123.5)	118.3 (113.1, 123.8)
A/South Australia/34/2019	Fluzone Quadrivalent (1286)	Day 0	38.3 (36.6, 40.1)	38.3 (36.6, 40.1)	38.3 (36.6, 40.1)
	Fluzone Quadrivalent (1284)	Day 28	70.4 (66.3, 74.7)	70.4 (66.3, 74.7)	70.4 (66.4, 74.7)
	Quad-NIV (1280)	Day 0	39.3 (37.5, 41.2)	39.2 (37.4, 41.1)	39.1 (37.3, 41.0)
		Day 28	98.1 (92.1, 104.4)	98.3 (92.3, 104.6)	97.7 (91.8, 104.1)
A/California/94/2019	Fluzone Quadrivalent (1286)	Day 0	44.0 (42.0, 46.0)	43.9 (41.9, 46.0)	43.9 (41.9, 46.0)
		Day 28	80.6 (75.9, 85.6)	80.4 (75.7, 85.5)	80.4 (75.7, 85.4)
	Quad-NIV (1280)	Day 0	44.5 (42.4, 46.7)	44.5 (42.4, 46.7)	44.6 (42.5, 46.8)
		Day 28	115.0 (108.0, 122.4)	114.7 (107.7, 122.1)	114.9 (107.9, 122.3)
A/Cardiff/0508/2019	Fluzone Quadrivalent (1286)	Day 0	25.7 (24.7, 26.6)	25.6 (24.7, 26.6)	25.7 (24.7, 26.7)
		Day 28	45.4 (43.1, 47.8)	45.5 (43.2, 47.9)	45.4 (43.1, 47.8)
	Quad-NIV (1280)	Day 0	27.0 (26.0, 28.1)	26.9 (25.9, 28.0)	27.0 (25.9, 28.1)
		Day 28	63.9 (60.5, 67.6)	63.8 (60.4, 67.5)	63.9 (60.5, 67.6)
A/Idaho/13/2018	Fluzone Quadrivalent (1286)	Day 0	52.9 (50.9, 55.0)	52.9 (50.9, 54.9)	52.9 (50.9, 55.0)
	Fluzone Quadrivalent (1283)	Day 28	136.8 (129.5, 144.6)	136.8 (129.4, 144.6)	136.7 (129.3, 144.6)
	Quad-NIV (1279)	Day 0	53.6 (51.6, 55.8)	53.8 (51.7, 55.9)	53.9 (51.8, 56.0)
		Day 28	202.5 (191.2, 214.4)	202.4 (191.2, 214.3)	202.2 (190.9, 214.2)
A/Netherlands 1268/2019	Fluzone Quadrivalent (1286)	Day 0	39.6 (38.0, 41.3)	39.7 (38.0, 41.4)	39.5 (37.8, 41.2)
	Fluzone Quadrivalent (1284)	Day 28	74.7 (70.6, 79.0)	74.7 (70.6, 79.1)	74.9 (70.8, 79.3)
	Quad-NIV (1280)	Day 0	39.4 (37.7, 41.2)	39.6 (37.8, 41.4)	39.4 (37.7, 41.2)
		Day 28	102.3 (96.5, 108.5)	102.1 (96.3, 108.4)	102.2 (96.4, 108.4)
A/Tokyo/EH1801/2018	Fluzone Quadrivalent (1286)	Day 0	31.2 (30.1, 32.3)	31.1 (30.1, 32.3)	31.3 (30.2, 32.4)
	Fluzone Quadrivalent (1284)	Day 28	54.5 (51.7, 57.4)	54.4 (51.6, 57.3)	54.6 (51.9, 57.5)
	Quad-NIV (1280)	Day 0	32.2 (31.0, 33.4)	32.3 (31.1, 33.5)	32.2 (31.0, 33.4)
		Day 28	78.0 (73.8, 82.5)	78.0 (73.8, 82.5)	78.1 (73.8, 82.6)
B/Washington/02/2019	Fluzone Quadrivalent (1286)	Day 0	48.4 (46.8, 50.1)	48.3 (46.7, 50.0)	48.3 (46.7, 50.0)
	Fluzone Quadrivalent (1283)	Day 28	71.4 (69.0, 74.0)	71.4 (68.9, 73.9)	71.4 (68.9, 74.0)
	Quad-NIV (1279)	Day 0	48.7 (47.0, 50.5)	49.0 (47.2, 50.8)	48.5 (46.8, 50.3)
		Day 28	88.2 (84.7, 91.8)	87.9 (84.3, 91.5)	88.3 (84.8, 91.9)

2019–2020 Fluzone^®^ Quadrivalent was used. Analysis details are included in the methods section. Geometric mean titer (GMT) was defined as the antilog of the mean of the log-transformed HAI titers for a given treatment group [23]. CI, confidence interval; GMT, geometric mean titer; HAI, hemagglutination inhibition; VLP, virus-like particle.

## Data Availability

The data presented in this study are available on request from the corresponding author. They are not publicly available due to proprietary subject and sample information.

## Supplementary Materials

The following supporting information can be downloaded at: https://www.mdpi.com/article/10.3390/microorganisms12112358/s1, Table S1: Source and details of human and animal serum samples tested in egg-derived and VLP HAI validation assays; Table S2: Results of linearity regression parameters of egg-derived virus HAI assay for four homologous seasonal influenza strains (A/Kansas, A/Brisbane, B/Maryland, and B/Phuket); Table S3: Results of linearity regression parameters of VLP HAI assay for four homologous seasonal influenza strains (A/Kansas, A/Brisbane, B/Maryland, and B/Phuket) and drifted strains (A/California, A/Cardiff, A/Netherlands, and A/Tokyo); Table S4: Accuracy (% Relative bias) and precision (%GCV) of the HAI titers linearity with A/Kansas/14/2017 egg-derived virus/VLP; Table S5: Accuracy (% Relative Bias) and precision (%GCV) of the HAI titers linearity with A/Brisbane/02/2018, B/Maryland/15/2016, B/Phuket/3073/2013, A/California/94/2019, A/Cardiff/0508/2019, A/Netherlands/1268/2019, and A/Tokyo/EH1801/2018 virus strains; Table S6: Assay robustness—effect of human RBC suspension storage time using four homologous seasonal influenza strains (A/Kansas, A/Brisbane, B/Maryland, and B/Phuket); Table S7: Assay robustness—effect of plate reading time (incubation time) using four homologous seasonal influenza strains (A/Kansas, A/Brisbane, B/Maryland, and B/Phuket); Table S8: Assay robustness in terms of HAI GMT % difference from baseline—effect of combined serum–egg-derived virus/VLP incubation time and plate reading time using four homologous seasonal influenza strains (A/Kansas, A/Brisbane, B/ Maryland, and B/Phuket); Table S9: Stability of RDE-treated samples in egg-derived and VLP HAI assay using four homologous seasonal influenza strains (A/Kansas, A/Brisbane, B/Maryland, and B/Phuket); Table S10: Total %GCV for singleton titers, paired replicates, and random replicates for A/Kansas/14/2017 VLP HAI assay; Table S11: Total %GCV for singleton titers, paired replicates, and random replicates for A/Brisbane/02/2018 VLP HAI assay; Table S12: Total %GCV for singleton titers, paired replicates, and random replicates for B/Maryland/15/2016 VLP HAI assay; Table S13: Total %GCV for singleton titers, paired replicates, and random replicates for B/Phuket/3073/2013 VLP HAI assay; Table S14: Comparison of CypherOne Readout with the manual assay readout; Figure S1: Correlation of Hemagglutination-Inhibition Titers Against A/Kansas/14/2017 in Clinical Trial Sera as Determined by Assays Using Wild-type VLPs and Egg-grown Viruses as Agglutinins.
