# Supplementary material for: A Modified Novel Validated High-Throughput Hemagglutinin Inhibition Assay Using Recombinant Virus-like Particles and Human Red Blood Cells for the Objective Evaluation of Recombinant Hemagglutinin Nanoparticle Seasonal Influenza Vaccine
Source: Microorganisms. 2024 Nov 19;12(11):2358. doi: 10.3390/microorganisms12112358 (PMC11596312; doi:10.3390/microorganisms12112358)
Supplement: Supplementary file 1 [file microorganisms-12-02358-s001.zip › microorganisms-3277320-supplementary.pdf]

microorganisms

MDPI

SUPPLEMENT:

Table S1. Source and details of human and animal serum samples tested in egg-derived virus and VLP HAI validation assays.

No.	Sample #	Serum type/source
1	Sample #1	Human serum/Valley Biomedical, anti-HA antibodies depleted in Novavax
2	Sample #2	Human serum/BioIVT, anti-HA antibodies depleted in Novavax
3	Sample #3	Human serum/BioIVT, screened HAI negative for at least 1 influenza strain
4	Sample #4	Human serum/BioIVT, anti-HA antibodies depleted in Novavax
5	Sample #5	Human serum/BioIVT, anti-HA antibodies depleted in Novavax
6	Sample #6	Human serum/BioIVT, anti-HA antibodies depleted in Novavax
7	Sample #7	Human serum/BioIVT, screened HAI positive for at least 1 influenza strain
8	Sample #8	Human serum/BioIVT, anti-HA antibodies depleted in Novavax
9	Sample #9	Human serum/BioIVT, anti-HA antibodies depleted in Novavax
10	Sample #10	Human serum/BioIVT, anti-HA antibodies depleted in Novavax
11	Sample #11	Human serum/BioIVT, anti-HA antibodies depleted in Novavax
12	Sample #12	Human serum/BioIVT, screened HAI positive for at least 1 influenza strain
13	Sample #13	Human serum/BioIVT, screened HAI positive for at least 1 influenza strain
14	Sample #14	Human serum/BioIVT, screened HAI positive for at least 1 influenza strain
15	Sample #15	Human serum/BioIVT, screened HAI positive for at least 1 influenza strain
16	Sample #16	Human serum/BioIVT, screened HAI positive for at least 1 influenza strain
17	Sample #17	Human serum/BioIVT, reported receipt of influenza vaccine within the 2017–2018 Flu season
18	Sample #18	Human serum/BioIVT, reported receipt of influenza vaccine within the 2017–2018 Flu season
19	Sample #19	Human serum/BioIVT, reported receipt of influenza vaccine within the 2017–2018 Flu season
20	Sample #20	Human serum/BioIVT, reported receipt of influenza vaccine within the 2017–2018 Flu season
21	Sample #21	Human serum/BioIVT, reported receipt of influenza vaccine within the 2017–2018 Flu season
22	Sample #22	Human serum/BioIVT, reported receipt of influenza vaccine within the 2017–2018 Flu season
23	Sample #23	Human serum/BioIVT reported receipt of influenza vaccine within the 2017–2018 Flu season
24	Sample #24	Human serum/BioIVT, reported receipt of influenza vaccine within the 2017–2018 Flu season

25	Sample #25	Human serum/BioIVT, reported receipt of influenza vaccine within the 2017–2018 Flu season
26	Sample #26	Human serum/BioIVT, reported receipt of influenza vaccine within the prior year
27	Sample #27	Sheep serum/NIBSC
28	Sample #28	Sheep serum/NIBSC
29	Sample #29	Sheep serum/NIBSC
30	Sample #30[1]	Sheep serum/NIBSC
31	Sample #31[2]	Sheep serum/NIBSC
32	Sample #32[2]	Sheep serum/NIBSC
33	Sample #33	Sheep serum/NIBSC
34	Sample #34	Human serum/Biological Specialty Co./BioIVT, reported receipt of influenza vaccine within the 2017–2018 Flu season
35	Sample #35	Human serum/Biological Specialty Co./BioIVT, reported receipt of influenza vaccine within the 2017–2018 Flu season
36	Sample #36	Human serum/Biological Specialty Co./BioIVT, reported receipt of influenza vaccine within the 2017–2018 Flu season
37	Sample #37	Human serum/Biological Specialty Co./BioIVT, reported receipt of influenza vaccine within the 2017–2018 Flu season
38	Sample #38	Human serum/Biological Specialty Co./BioIVT, reported receipt of influenza vaccine within the 2017–2018 Flu season
39	Sample #39	Human serum/Biological Specialty Co./BioIVT, reported receipt of influenza vaccine within the 2017–2018 Flu season
40	Sample #40	Human serum/Biological Specialty Co./BioIVT, reported receipt of influenza vaccine within the 2017–2018 Flu season
41	Sample #41	Sheep serum/Novavax
42	Sample #42[1]	Sheep serum/Novavax
43	Sample #43	Sheep serum/Novavax
44	Sample #44	Sheep serum /Novavax (Study NVX 733-025, Day 63 serum)
45	Sample #45	Sheep serum /Novavax (Study NVX 733-025, Day 63 serum)
46	Sample #46[3]	Sheep serum /Novavax (Study NVX 733-027, Day 44 serum)
47	Sample #47	Sheep serum /Novavax (Study NVX 733-027, Day 44 serum)
48	Sample #48	Sheep serum /Novavax (Study NVX 733-027, Day 45 serum)
49	Sample #49	Sheep serum /Novavax (Study NVX 733-027, Day 44 serum)
50	Sample #50[3]	Sheep serum /Novavax (Study NVX 733-031, Day 45 serum)
51	Sample #51[3]	Sheep serum /Novavax (Study NVX 733-031, Day 45 serum)
52	Sample #52[3]	Sheep serum /Novavax (Study NVX 733-034, Day 35 serum)
53	Sample #53[3]	Sheep serum /Novavax (Study NVX 733-034, Day 35 serum)

[1]Samples used only in the egg-derived virus HAI assay. [2]Samples used in the VLP HAI assay: NIBSC Influenza anti-B/Brisbane/60/2008-HA Serum, Code 15/312; NIBSC Influenza Anti-B/Colorado/06/2017-Like HA Serum, Code 18/170. [3]Samples used only in the VLP HAI assay.
HA, hemagglutinin; HAI, hemagglutination inhibition; NIBSC, National Institute for Biological Standards and Control; VLP, virus-like particle.

Table S2. Results of linearity regression parameters of egg-derived virus HAI assay for four homologous seasonal influenza strains (A/Kansas, A/Brisbane, B/Maryland, and B/Phuket).

Strain	Sample	Parameter	Estimate	95% LCL	95% UCL
A/Kansas/14/2017	Sample #42	Slope	1.004	0.883	1.125
		Intercept	0.001	−0.203	0.205
		Residual variability (%GSD)	0.036 (8.7)	N/A	
		R^2	0.9957	N/A	
	Sample #43	Slope	1.132	0.976	1.289
		Intercept	−0.281	−0.585	0.022
		Residual variability (%GSD)	0.097 (25.0)	N/A	
		R^2	0.9857	N/A	
	Sample #47	Slope	0.975	0.841	1.109
		Intercept	0.099	−0.195	0.393
		Residual variability (%GSD)	0.107 (27.9)	N/A	
		R^2	0.9814	N/A	
A/Brisbane/02/2018	Sample #15	Slope	0.997	0.980	1.013
		Intercept	0.018	−0.017	0.054
		Residual variability (%GSD)	0.013 (3.1)	N/A	
		R^2	0.9997	N/A	
	Sample #49	Slope	1.041	0.902	1.180
		Intercept	−0.018	−0.333	0.296
		Residual variability (%GSD)	0.111 (29.1)	N/A	
		R^2	0.9824`	N/A	
B/Maryland/15/2016	Sample #14	Slope	0.975	0.937	1.012
		Intercept	0.066	−0.009	0.141
		Residual variability (%GSD)	0.023 (5.5)	N/A	
		R^2	0.9989	N/A	
	Sample #35	Slope	0.899	0.829	0.968
		Intercept	0.353	0.208	0.499

		Residual variability (%GSD)	0.043 (10.4)	N/A	
		R^2	0.9955	N/A	
B/Phuket/3073/2013	Sample #14	Slope	0.921	0.880	0.961
		Intercept	0.240	0.154	0.325
		Residual variability (%GSD)	0.032 (7.6)	N/A	
		R^2	0.9981	N/A	
	Sample #16	Slope	0.993	0.983	1.003
		Intercept	0.036	0.015	0.057
		Residual variability (%GSD)	0.008 (1.8)	N/A	
		R^2	0.9999	N/A	

%GSD, percent geometric standard deviation; HAI, hemagglutination inhibition; LCL, lower confidence limit; N/A, not applicable; R^2, coefficient of determination; UCL, upper confidence limit.

Table S3. Results of linearity regression parameters of VLP HAI assay for four homologous seasonal influenza strains (A/Kansas, A/Brisbane, B/Maryland, and B/Phuket) and drifted strains (A/California, A/Cardiff, A/Netherlands, and A/Tokyo).

Strain	Sample	Parameter	Estimate	95% LCL	95% UCL
A/Kansas/14/2017	Sample #46	Slope	0.991	0.942	1.040
		Intercept	0.049	−0.066	0.164
		Residual variability (%GSD)	0.049 (11.8)	N/A	
		R^2	0.9969	N/A	
	Sample #47	Slope	0.999	0.990	1.008
		Intercept	0.006	−0.015	0.027
		Residual variability (%GSD)	0.009 (2.1)	N/A	
		R^2	0.9999	N/A	
A/Brisbane/02/2018	Sample #15	Slope	0.986	0.960	1.012
		Intercept	0.075	0.019	0.130
		Residual variability (%GSD)	0.021 (5.0)	N/A	
		R^2	0.9993	N/A	
	Sample #49 (1:2 diluted)	Slope	1.008	0.970	1.047
		Intercept	−0.000	−0.084	0.084
		Residual variability (%GSD)	0.031 (7.3)	N/A	
		R^2	0.9985	N/A	
B/Maryland/15/2016	Sample #14	Slope	1.071	0.957	1.186
		Intercept	−0.261	−0.487	−0.036
		Residual variability (%GSD)	0.052 (12.7)	N/A	
		R^2	0.9941	N/A	
	Sample #45 (1:2 diluted)	Slope	0.996	0.973	1.018
		Intercept	0.011	−0.038	0.060
		Residual variability (%GSD)	0.018 (4.2)	N/A	
		R^2	0.9995	N/A	
B/Phuket/3073/2013	Sample #15	Slope	1.036	0.964	1.107
		Intercept	−0.063	−0.205	0.078
		Residual variability (%GSD)	0.044 (10.8)	N/A	

		R^2	0.9964	N/A	
	Sample #16	Slope	1.140	1.019	1.261
		Intercept	−0.331	−0.573	−0.089
		Residual variability (%GSD)	0.075 (18.9)	N/A	
		R^2	0.9915	N/A	
A/California/94/2019	Sample #43 (1:4 diluted)	Slope	0.932	0.907	0.957
		Intercept	0.249	0.191	0.306
		Residual variability (%GSD)	0.025 (5.9)	N/A	
		R^2	0.9991	N/A	
	Sample #50 (1:4 diluted)	Slope	0.955	0.884	1.026
		Intercept	0.172	0.018	0.325
		Residual variability (%GSD)	0.044 (10.6)	N/A	
		R^2	0.9959	N/A	
A/Cardiff/0508/2019	Sample #43	Slope	1.014	0.894	1.133
		Intercept	0.031	−0.228	0.290
		Residual variability (%GSD)	0.095 (24.6)	N/A	
		R^2	0.9862	N/A	
	Sample #53	Slope	0.957	0.863	1.052
		Intercept	0.233	0.025	0.441
		Residual variability (%GSD)	0.076 (19.0)	N/A	
		R^2	0.9903	N/A	
A/Nether-lands/1268/2019	Sample #43 (1:2 diluted)	Slope	0.978	0.892	1.063
		Intercept	0.126	−0.061	0.312
		Residual variability (%GSD)	0.053 (13.0)	N/A	
		R^2	0.9942	N/A	
	Sample #52	Slope	0.992	0.972	1.011
		Intercept	0.041	−0.001	0.082
		Residual variability (%GSD)	0.015 (3.6)	N/A	
		R^2	0.9996	N/A	
A/Tokyo/EH1801/2018	Sample #43 (1:2 diluted)	Slope	1.005	0.977	1.033
		Intercept	−0.007	−0.068	0.053

		Residual variability (%GSD)	0.022 (5.3)	N/A	
		R^2	0.9992	N/A	
	Sample #51 (1:2 diluted)	Slope	0.966	0.798	1.133
		Intercept	0.171	−0.172	0.513
		Residual variability (%GSD)	0.104 (27.0)	N/A	
		R^2	0.9777	N/A	

%GSD, percent geometric standard deviation; HAI, hemagglutination inhibition; LCL, lower confidence limit; N/A, not applicable; R^2, coefficient of determination; UCL, upper confidence limit; VLP, virus-like particle.

Table S4. Accuracy (% Relative bias) and precision (%GCV) of the HAI titers linearity with A/Kansas/14/2017 egg-derived virus/VLP.

		**Dilution**	**1**	**2**	**4**	**8**	**16**	**1**	**2**	**4**	**8**	**16**	**32**	**64**	**1**	**2**	**4**	**8**	**16**	**32**	**64**	**128**
A/Kansas/14/2017			Sample #42					Sample #43							Sample #47							
	Egg-derived	% Relative bias	−0.0	−2.8	15.5	0.0	−2.8	0.0	−0.0	15.5	15.5	9.1	−35.2	−42.2	0.0	−5.6	−5.6	18.9	58.7	58.7	−5.6	−5.6
		Total %GCV	20.5	10.2	31.4	20.5	9.9	33.7	36.8	0.0	0.0	20.2	33.7	0.0	27.1	0.0	0.0	31.7	28.2	34.0	0.0	0.0

			**1**	**2**	**4**	**8**	**16**	**32**	**64**	**128**	**256**	**1**	**2**	**4**	**8**	**16**	**32**	**64**	**128**	**256**
A/Kansas/14/2017			Sample #46									Sample #47								
	VLP	% Relative bias	0.0	0.0	0.0	5.9	33.5	22.4	0.0	0.0	5.9	0.0	0.0	0.0	0.0	0.0	5.9	0.0	0.0	0.0
		Total %GCV	0.0	0.0	0.0	20.5	36.0	34.0	0.0	0.0	20.5	0.0	0.0	0.0	0.0	0.0	20.5	0.0	0.0	0.0

$$\% \text{ Relative bias} = 100 \times \frac{(\text{Observed overall HAI GMT-Expected HAI GMT})}{\text{Expected HAI GMT}}$$

%GCV, percent geometric coefficient of variation; GMT, geometric mean titer; HAI, hemagglutination inhibition; VLP, virus-like particle.

Table S5. Accuracy (% Relative Bias) and precision (%GCV) of the HAI titers linearity with A/Brisbane/02/2018, B/Maryland/15/2016, B/Phuket/3073/2013, A/California/94/2019, A/Cardiff/0508/2019, A/Netherlands/1268/2019, and A/Tokyo/EH1801/2018 virus strains.

	**Dilution**		**1**	**2**	**4**	**8**	**16**	**32**	**64**	**128**	**256**	**1**	**2**	**4**	**8**	**16**	**32**	**64**	**128**
A/Brisbane/02/2018			Sample #15									Sample #49							
	Egg-derived	% Relative bias	0.0	0.0	9.1	−0.0	2.9	2.9	2.9	2.9	–	0.0	12.2	41.4	41.4	54.2	45.6	−10.9	−18.3
		Total %GCV	10.2	10.2	13.6	22.1	0.0	0.0	0.0	0.0	–	34.4	34.0	22.8	27.5	10.2	21.7	17.5	9.9
			Sample #15									Sample #49 (1:2 diluted)							
	VLP	% Relative bias	0.0	9.1	15.5	15.5	15.5	15.5	9.1	12.2	–	0.0	0.0	12.2	18.9	2.9	0.0	0.0	0.0
		Total %GCV	30.7	20.5	0.0	0.0	0.0	0.0	20.2	10.5	–	0.0	0.0	27.5	28.2	10.2	0.0	0.0	0.0
B/Maryland/15/2016			Sample #14									Sample #35							
	Egg-derived	% Relative bias	0.0	2.9	0.0	0.0	12.2	0.0	15.5	–	–	0.0	26.0	41.4	58.7	58.7	58.7	58.7	–
		Total %GCV	0.0	10.2	0.0	0.0	31.8	0.0	23.2	–	–	27.5	35.9	22.9	0.0	0.0	0.0	0.0	–
			Sample #14									Sample #45 (1:2 diluted)							
	VLP	% Relative bias	0.0	−29.3	−29.3	−29.3	−29.3	−29.3	–	–	–	0.0	0.0	−5.6	−0.0	9.1	0.0	0.0	0.0
		Total %GCV	37.7	0.0	0.0	0.0	0.0	0.0	–	–	–	0.0	0.0	20.5	32.8	44.0	0.0	0.0	0.0
B/Phuket/3073/2013	Egg-derived		Sample #14									Sample #16							
		% Relative Bias	0.0	9.1	15.5	15.5	15.5	15.5	49.8	54.2	–	0.0	5.9	5.9	5.9	5.9	5.9	5.9	5.9

		Total %GCV	26.1	20.5	0.0	0.0	0.0	0.0	39.3	41.5	–	20.5	0.0	0.0	0.0	0.0	0.0	0.0	0.0
	VLP		Sample #15									Sample #16							
		% Relative bias	0.0	5.9	5.9	5.9	5.9	5.9	−20.6	–	–	0.0	0.0	0.0	0.0	0.0	−33.3	−47.0	–
		Total %GCV	20.5	0.0	0.0	0.0	0.0	0.0	49.3	–	–	0.0	0.0	0.0	0.0	0.0	54.5	20.5	–
A/California/94/2019	VLP		Sample #43 (1:4 diluted)									Sample #50 (1:4 diluted)							
		% Relative bias	0.0	18.9	18.9	26.0	22.4	22.4	37.4	45.6	63.4	0.0	15.5	18.9	33.5	33.5	9.1	33.5	–
		Total %GCV	47.3	10.2	10.2	10.2	0.0	0.0	31.8	34.9	37.1	44.9	28.8	31.8	0.0	16.2	49.0	0.0	–
A/Cardiff/0508/2019			Sample #43									Sample #53							
		% Relative bias	0.0	2.9	22.4	54.2	58.7	−2.8	0.0	0.0	–	0.0	26.0	54.2	49.8	83.4	41.4	45.6	29.7
		Total %GCV	0.0	10.0	32.2	39.3	40.0	10.5	0.0	0.0	–	15.6	36.1	35.9	40.0	10.2	41.5	39.3	43.0
A/Netherlands/1268/2019			Sample #43 (1:2 diluted)									Sample #52							
		% Relative bias	0.0	22.4	29.7	29.7	29.7	2.9	29.7	–	–	0.0	2.9	5.9	9.1	12.2	2.9	5.9	5.9
		Total %GCV	32.4	20.5	0.0	0.0	0.0	40.0	0.0	–	–	20.5	10.2	0.0	10.2	15.5	10.5	0.0	0.0
A/Tokyo/EH1801/2018			Sample #43 (1:2 diluted)									Sample #51 (1:2 diluted)							
		% Relative bias	0.0	0.0	0.0	12.2	0.0	−5.6	0.0	0.0	–	0.0	12.2	41.4	63.4	63.4	−5.6	33.5	–
		Total %GCV	0.0	0.0	0.0	29.1	0.0	20.5	0.0	0.0	–	33.7	32.5	41.1	22.6	22.5	20.5	37.5	–

$$\% \text{ Relative bias} = 100 \times \frac{(\text{Observed overall HAI GMT-Expected HAI GMT})}{\text{Expected HAI GMT}}$$

%GCV, percent geometric coefficient of variation; GMT, geometric mean titer; HAI, hemagglutination inhibition; VLP, virus-like particle.

Table S6. Assay robustness – effect of human RBC suspension storage time using four homologous seasonal influenza strains (A/Kansas, A/Brisbane, B/Maryland, and B/Phuket).

**Strain**	**HAI assay type**	**% Difference from baseline[1]**	
		**0.75% RBC stored for 2 weeks**	**0.75% RBC from 10% RBC stored for 2 weeks**
A/Kansas/14/2017	Egg-derived	−50.0–88.8	−5.6–100.0
	VLP	−52.8–0.0	−50.0–100.0
A/Brisbane/02/2018	Egg-derived	−50.0–2.9	−29.3–100.0
	VLP	−50.0–2.9	−50.0–15.5
B/Maryland/15/2016	Egg-derived	−50.0–9.1	−48.5–100.0
	VLP	−50.0–100.0	−29.3–88.8
B/Phuket/3073/2013	Egg-derived	−50.0–33.5	−50.0–5.9
	VLP	−64.6–0.0	−50.0–0.0

$$\% \text{ Difference} = 100 \times \frac{(\text{Testing HAI GMT -Baseline Overall HAI GMT})}{\text{Baseline Overall HAI GMT}}$$

[1]Baseline values were overall HAI GMT from the precision assay runs utilizing fresh 0.75% RBCs stored at 2 to 8 °C for less than a week (≤7 days).

GMT, geometric mean titer; HAI, hemagglutination inhibition; RBC, red blood cells; VLP, virus-like particle.

Table S7. Assay robustness – effect of plate reading time (incubation time) using four homologous seasonal influenza strains (A/Kansas, A/Brisbane, B/Maryland, and B/Phuket).

Strain	HAI assay type	% Difference (Range) at varying plate reading time relative to standard 90 min		
		75 min	120 min	150 min
A/Kansas/14/2017	Egg-derived	−29.3–0.0	0.0–41.4	0.0–100.0
	VLP	−29.3–0.0	0.0–68.2	0.0–100.0
A/Brisbane/02/2018	Egg-derived	0.0–41.4	0.0–41.4	0.0–41.4
	VLP	−40.5–0.0	0.0–68.2	0.0–100.0
B/Maryland/15/2016	Egg-derived	−15.9–41.4	0.0–68.2	0.0–100.0
	VLP	−15.9–41.4	0.0–41.4	0.0–41.4
B/Phuket/3073/2013	Egg-derived	−15.9–41.4	0.0–41.4	0.0–41.4
	VLP	−15.9–0.0	0.0–68.2	0.0–68.2

$$\text{\% Difference}=100\times\frac{\text{(Testing HAI GMT -Baseline Overall HAI GMT)}}{\text{Baseline Overall HAI GMT}}$$

GMT, geometric mean titer; HAI, hemagglutination inhibition; VLP, virus-like particle.

Table S8. Assay robustness in terms of HAI GMT % difference from baseline – effect of combined serum–egg-derived virus/VLP incubation time and plate reading time using four homologous seasonal influenza strains (A/Kansas, A/Brisbane, B/Maryland, and B/Phuket).

**Serum-virus/VLP incubation time**	**Plate reading time (min)**	**A/Kansas/14/2017**		**A/Brisbane/02/2018**		**B/Maryland/15/2016**		**B/Phuket/3073/2013**	
		Egg-derived	VLP	Egg-derived	VLP	Egg-derived	VLP	Egg-derived	VLP
50 min	75	−50.0–0.0	−50.0–41.4	−50.0–0.0	−50.0–41.4	−50.0–0.0	−50.0–0.0	−50.0–0.0	−50.0–0.0
	90	−50.0–0.0	−50.0–41.4	−50.0–0.0	−50.0–41.4	−50.0–0.0	−50.0–0.0	−50.0–41.4	−50.0–0.0
	120	−50.0–0.0	−50.0–100.0	−50.0–41.4	−50.0–41.4	−50.0–0.0	−50.0–0.0	−50.0–41.4	−50.0–0.0
	150	−50.0–0.0	−50.0–100.0	−50.0–41.4	−50.0–41.4	−50.0–0.0	−50.0–0.0	−50.0–100.0	−50.0–0.0
60 min	75	−50.0–0.0	−29.3–0.0	0.0–41.4	−50.0–0.0	−50.0–0.0	−50.0–0.0	−50.0–0.0	−50.0–0.0
	90	–	–	–	–	–	–	–	–
	120	0.0–100.0	0.0–100.0	0.0–100.0	0.0	0.0–100.0	0.0	0.0–100.0	0.0–41.4
	150	0.0–100.0	0.0–100.0	0.0–100.0	0.0–41.4	0.0–100.0	0.0–100.0	0.0–100.0	0.0–41.4
70 min	75	−50.0–100.0	−50.0–100.0	−29.3–100.0	−50.0–100.0	−29.3–100.0	0.0–100.0	−50.0–100.0	−29.3–41.4
	90	−29.3–100.0	−50.0–100.0	0.0–100.0	−50.0–100.0	0.0–100.0	0.0–100.0	0.0–100.0	0.0–41.4
	120	−29.3–100.0	−50.0–100.0	0.0–100.0	−50.0–100.0	0.0–100.0	0.0–100.0	0.0–100.0	0.0–100.0
	150	−29.3–100.0	−50.0–100.0	0.0–100.0	−50.0–100.0	0.0–100.0	0.0–100.0	0.0–100.0	0.0–100.0

$$\% \text{ Difference} = 100 \times \frac{(\text{Testing HAI GMT -Baseline Overall HAI GMT})}{\text{Baseline Overall HAI GMT}}$$

Geometric mean titer (GMT) was defined as the antilog of the mean of the log-transformed HAI titers for a given treatment group [21].

*Baseline HAI GMTs were from assay runs in which serum-virus/VLP incubation time was 1 h and the plate reading time was 90 min after RBC addition.

GMT, geometric mean titer; HAI, hemagglutination inhibition; VLP, virus-like particle.

Table S9. Stability of RDE-treated samples in egg-derived and VLP HAI assay using four homologous seasonal influenza strains (A/Kansas, A/Brisbane, B/Maryland, and B/Phuket).

Strain	HAI assay type	% Difference from baseline[1]			
		Stored at 2 to 8 °C for 1 month	Stored at 2 to 8 °C for 2 months	Stored at ≤−20 °C for 2 months	Two freeze/thaw cycles
A/Kansas/14/2017	Egg-derived	−52.8–78.2	−75.0–0.0	−5.6–535.0	−42.2–100.0
	VLP	−70.3–41.4	−70.3–100.0	−50.0–100.0	−5.6–100.0
A/Brisbane/02/2018	Egg-derived	−50.0–94.3	−50.0–58.7	−50.0–100.0	−50.0–88.8
	VLP	−56.7–0.0	−56.7–78.2	−50.0–100.0	−29.3–78.2
B/Maryland/15/2016	Egg-derived	−50.0–41.4	−50.0–100.0	−20.6–182.8	−43.9–100.0
	VLP	−50.0–41.4	−50.0–88.8	−75.0–49.8	−25.1–41.4
B/Phuket/3073/2013	Egg-derived	−50.0–88.8	−50.0–15.5	−35.2–100.0	−33.3–100.0
	VLP	−50.0–94.3	−50.0–0.0	−29.3–100.0	−50.0–41.4

$$\%\ \text{Difference}=100\times\frac{(\text{Testing HAI GMT -Baseline Overall HAI GMT})}{\text{Baseline Overall HAI GMT}}$$

[1]Baseline values were overall HAI GMT results from the precision assay runs in which RDE-treated samples were stored at 2 to 8 °C for ≤7 days before testing.

GMT, geometric mean titer; HAI, hemagglutination inhibition; RDE, receptor-destroying enzyme; VLP, virus-like particle.

Table S10. Total %GCV for singleton titers, paired replicates, and random replicates for A/Kansas/14/2017 VLP HAI assay.

No.	Sample ID	%GCV				% Difference		
		**Singleton titers**	**Paired replicates**	**Random replicates 1**	**Random replicates 2**	**Singleton titers vs. Paired replicates**	**Random 1 vs. Paired replicates**	**Random 2 vs. Paired replicates**
1	Sample #3	0.0	0.0	0.0	0.0	0.0	0.0	0.0
2	Sample #3 (1:2)	0.0	0.0	0.0	0.0	0.0	0.0	0.0
3	Sample #15	0.0	0.0	0.0	0.0	0.0	0.0	0.0
4	Sample #16	21.7	20.5	20.5	20.5	5.4	0.0	0.0
5	Sample #19	0.0	0.0	0.0	0.0	0.0	0.0	0.0
6	Sample #20	35.3	34.9	34.9	34.9	1.3	0.0	0.0
7	Sample #21	35.5	35.9	35.9	35.9	−1.0	0.0	0.0
8	Sample #23	21.7	20.5	20.5	20.5	5.4	0.0	0.0
9	Sample #24	36.2	32.2	37.0	42.8	12.4	14.8	32.8
10	Sample #25	0.0	0.0	0.0	0.0	0.0	0.0	0.0
11	Sample #26	37.5	35.6	38.6	36.9	5.4	8.4	3.6
12	Sample #34	0.0	0.0	0.0	0.0	0.0	0.0	0.0
13	Sample #35	0.0	0.0	0.0	0.0	0.0	0.0	0.0
14	Sample #37	0.0	0.0	0.0	0.0	0.0	0.0	0.0
15	Sample #39	0.0	0.0	0.0	0.0	0.0	0.0	0.0

16	Sample #43	14.3	10.2	20.5	20.5	40.4	101.6	101.6
17	Sample #46 Dil. 1	0.0	0.0	0.0	0.0	0.0	0.0	0.0
18	Sample #46 Dil. 2	0.0	0.0	0.0	0.0	0.0	0.0	0.0
19	Sample #46 Dil. 3	21.7	20.5	0.0	0.0	5.4	−100.0	−100.0
20	Sample #46 Dil. 4	38.9	36.0	20.5	20.5	8.1	−42.9	−42.9
21	Sample #46 Dil. 5	38.6	34.0	41.5	37.5	13.3	21.8	10.2
22	Sample #46 Dil. 6	0.0	0.0	34.9	40.0	0.0	0.0	0.0
23	Sample #46 Dil. 7	0.0	0.0	0.0	0.0	0.0	0.0	0.0
24	Sample #46 Dil. 8	21.7	20.5	0.0	0.0	5.4	−100.0	−100.0
25	Sample #46 Dil. 9	39.9	40.0	20.5	20.5	−0.3	−48.6	−48.6
26	Sample #46 Dil. 10	0.0	0.0	40.0	40.0	0.0	0.0	0.0
27	Sample #47 Dil. 1	0.0	0.0	0.0	0.0	0.0	0.0	0.0
28	Sample #47 Dil. 2	0.0	0.0	0.0	0.0	0.0	0.0	0.0
29	Sample #47 Dil. 3	0.0	0.0	0.0	0.0	0.0	0.0	0.0
30	Sample #47 Dil. 4	0.0	0.0	0.0	0.0	0.0	0.0	0.0
31	Sample #47 Dil. 5	21.7	20.5	0.0	0.0	5.4	−100.0	−100.0
32	Sample #47 Dil. 6	0.0	0.0	20.5	20.5	0.0	0.0	0.0
33	Sample #47 Dil. 7	0.0	0.0	0.0	0.0	0.0	0.0	0.0

34	Sample #47 Dil. 8	0.0	0.0	0.0	0.0	0.0	0.0	0.0
35	Sample #47 Dil. 9	0.0	0.0	0.0	0.0	0.0	0.0	0.0
36	Sample #47 Dil. 10	0.0	0.0	0.0	0.0	0.0	0.0	0.0

$$\% \text{ Difference} = 100 \times \frac{(\text{Paired or Random }\%\text{GCV-Singleton }\%\text{GCV})}{\text{Singleton }\%\text{GCV}}$$

%GCV, percent geometric coefficient of variation; HAI, hemagglutination inhibition; VLP, virus-like particle.

Table S11. Total %GCV for singleton titers, paired replicates, and random replicates for A/Brisbane/02/2018 VLP HAI assay.

No.	Sample ID	%GCV				% Difference		
		Singleton titers	Paired replicates	Random replicates 1	Random replicates 2	Singleton titers vs. Paired replicates	Random 1 vs. Paired replicates	Random 2 vs. Paired replicates
1	Sample #3	34.6	33.7	33.7	33.7	2.6	0.0	0.0
2	Sample #3 (1:2)	0.0	0.0	0.0	0.0	0.0	0.0	0.0
3	Sample #13	19.8	13.6	20.5	20.5	45.8	51.6	51.6
4	Sample #14	0.0	0.0	0.0	0.0	0.0	0.0	0.0
5	Sample #15 Dil. 1	33.4	30.7	33.0	33.7	8.6	7.5	9.7
6	Sample #15 Dil. 2	21.7	20.5	20.5	20.5	5.4	0.0	0.0
7	Sample #15 Dil. 3	0.0	0.0	0.0	0.0	0.0	0.0	0.0
8	Sample #15 Dil. 4	0.0	0.0	0.0	0.0	0.0	0.0	0.0
9	Sample #15 Dil. 5	0.0	0.0	0.0	0.0	0.0	0.0	0.0
10	Sample #15 Dil. 6	0.0	0.0	0.0	0.0	0.0	0.0	0.0
11	Sample #15 Dil. 7	21.1	20.2	20.2	20.2	4.5	0.0	0.0
12	Sample #15 Dil. 8	14.3	10.2	0.0	0.0	40.4	−100.0	−100.0
13	Sample #15 Dil. 9	0.0	0.0	0.0	0.0	0.0	0.0	0.0
14	Sample #17	0.0	0.0	0.0	0.0	0.0	0.0	0.0
15	Sample #18	30.0	28.4	32.1	32.1	5.7	13.0	13.0

16	Sample #19	32.5	26.7	44.3	27.5	21.5	65.8	2.9
17	Sample #20	0.0	0.0	0.0	0.0	0.0	0.0	0.0
18	Sample #22	0.0	0.0	0.0	0.0	0.0	0.0	0.0
19	Sample #23	14.2	10.0	20.2	20.2	41.8	101.5	101.5
20	Sample #24	0.0	0.0	0.0	0.0	0.0	0.0	0.0
21	Sample #25	0.0	0.0	0.0	0.0	0.0	0.0	0.0
22	Sample #26	0.0	0.0	0.0	0.0	0.0	0.0	0.0
23	Sample #49 Dil. 1	0.0	0.0	0.0	0.0	0.0	0.0	0.0
24	Sample #49 Dil. 2	0.0	0.0	0.0	0.0	0.0	0.0	0.0
25	Sample #49 Dil. 3	26.9	27.5	27.5	27.5	−2.3	0.0	0.0
26	Sample #49 Dil. 4	31.5	28.2	32.1	35.9	11.7	14.0	27.3
27	Sample #49 Dil. 5	14.3	10.2	20.5	20.5	40.4	101.6	101.6
28	Sample #49 Dil. 6	0.0	0.0	0.0	0.0	0.0	0.0	0.0
29	Sample #49 Dil. 7	0.0	0.0	0.0	0.0	0.0	0.0	0.0
30	Sample #49 Dil. 8	0.0	0.0	0.0	0.0	0.0	0.0	0.0
31	Sample #49 Dil. 9	30.0	24.2	32.1	35.9	24.3	33.0	48.5
32	Sample #49 Dil. 10	0.0	0.0	0.0	0.0	0.0	0.0	0.0
33	Sample #37	50.7	48.7	54.0	54.0	4.1	11.0	11.0

34	Sample #38	0.0	0.0	0.0	0.0	0.0	0.0	0.0
35	Sample #39	21.7	16.5	0.0	0.0	31.2	−100.0	−100.0
36	Sample #48	0.0	0.0	0.0	0.0	0.0	0.0	0.0

$$\% \text{ Difference} = 100 \times \frac{(\text{Paired or Random } \%\text{GCV-Singleton } \%\text{GCV})}{\text{Singleton } \%\text{GCV}}$$

%GCV, percent geometric coefficient of variation; HAI, hemagglutination inhibition; VLP, virus-like particle.

Table S12. Total %GCV for singleton titers, paired replicates, and random replicates for B/Maryland/15/2016 VLP HAI assay.

No.	Sample ID	%GCV				% Difference		
		Singleton titers	Paired replicates	Random replicates 1	Random replicates 2	Singleton titers vs. Paired replicates	Random 1 vs. Paired replicates	Random 2 vs. Paired replicates
1	Sample #3	0.0	0.0	0.0	0.0	0.0	0.0	0.0
2	Sample #3 (1:2)	0.0	0.0	0.0	0.0	0.0	0.0	0.0
3	Sample #7	40.4	37.1	40.0	41.5	8.8	7.6	11.7
4	Sample #12	32.8	31.8	31.8	31.8	3.1	0.0	0.0
5	Sample #13	0.0	0.0	0.0	0.0	0.0	0.0	0.0
6	Sample #14 Dil. 1	40.5	37.7	39.2	42.0	7.6	4.0	11.4
7	Sample #14 Dil. 2	0.0	0.0	0.0	0.0	0.0	0.0	0.0
8	Sample #14 Dil. 3	0.0	0.0	0.0	0.0	0.0	0.0	0.0
9	BRH1452659 Dil. 4 (Sample #14)	0.0	0.0	0.0	0.0	0.0	0.0	0.0
10	Sample #14 Dil. 5	0.0	0.0	0.0	0.0	0.0	0.0	0.0
11	Sample #14 Dil. 6	0.0	0.0	0.0	0.0	0.0	0.0	0.0
12	Sample #14 Dil. 7	56.0	55.4	55.4	55.4	1.1	0.0	0.0
13	Sample #15	31.7	30.1	28.7	34.9	5.4	−4.7	15.9
14	Sample #16	0.0	0.0	0.0	0.0	0.0	0.0	0.0
15	Sample #17	0.0	0.0	0.0	0.0	0.0	0.0	0.0

16	Sample #19	27.1	24.4	20.2	31.8	10.9	−17.3	29.9
17	Sample #21	0.0	0.0	0.0	0.0	0.0	0.0	0.0
18	Sample #22	35.5	32.4	34.9	35.9	9.4	7.5	10.6
19	Sample #23	0.0	0.0	0.0	0.0	0.0	0.0	0.0
20	Sample #24	0.0	0.0	0.0	0.0	0.0	0.0	0.0
21	Sample #26	0.0	0.0	0.0	0.0	0.0	0.0	0.0
22	Sample #45 Dil. 1	0.0	0.0	0.0	0.0	0.0	0.0	0.0
23	Sample #45 Dil. 2	0.0	0.0	0.0	0.0	0.0	0.0	0.0
24	Sample #45 Dil. 3	21.7	20.5	20.5	20.5	5.4	0.0	0.0
25	Sample #45 Dil. 4	36.4	32.9	35.3	20.5	10.8	7.2	−37.5
26	Sample #45 Dil. 5	48.3	44.1	61.0	46.8	9.6	38.3	6.3
27	Sample #45 Dil. 6	0.0	0.0	0.0	0.0	0.0	0.0	0.0
28	Sample #45 Dil. 7	0.0	0.0	0.0	0.0	0.0	0.0	0.0
29	Sample #45 Dil. 8	0.0	0.0	0.0	0.0	0.0	0.0	0.0
30	Sample #45 Dil. 9	21.7	20.5	20.5	20.5	5.4	0.0	0.0
31	Sample #45 Dil. 10	0.0	0.0	0.0	0.0	0.0	0.0	0.0
32	Sample #36	14.2	10.0	0.0	0.0	0.0	0.0	0.0
33	Sample #37	0.0	0.0	0.0	0.0	0.0	0.0	0.0

34	Sample #38	0.0	0.0	0.0	0.0	0.0	0.0	0.0
35	Sample #39	0.0	0.0	0.0	0.0	0.0	0.0	0.0
36	Sample #44	38.3	37.1	37.5	39.7	3.3	1.2	7.1

$$\%\ \text{Difference} = 100 \times \frac{(\text{Paired or Random }\%\text{GCV-Singleton }\%\text{GCV})}{\text{Singleton }\%\text{GCV}}$$

%GCV, percent geometric coefficient of variation; HAI, hemagglutination inhibition; VLP, virus-like particle.

Table S13. Total %GCV for singleton titers, paired replicates, and random replicates for B/Phuket/3073/2013 VLP HAI assay.

No.	Sample ID	%GCV				%Difference		
		**Singleton titers**	**Paired replicates**	**Random replicates 1**	**Random replicates 2**	**Singleton titers vs. Paired replicates**	**Random 1 vs. Paired replicates**	**Random 2 vs. Paired replicates**
1	Sample #3	0.0	0.0	0.0	0.0	0.0	0.0	0.0
2	Sample #3 (1:2)	0.0	0.0	0.0	0.0	0.0	0.0	0.0
3	Sample #13	0.0	0.0	0.0	0.0	0.0	0.0	0.0
4	Sample #14	21.7	20.5	20.5	20.5	5.4	0.0	0.0
5	Sample #15 Dil. 1	21.7	20.5	20.5	20.5	5.4	0.0	0.0
6	Sample #15 Dil. 2	0.0	0.0	0.0	0.0	0.0	0.0	0.0
7	Sample #15 Dil. 3	0.0	0.0	0.0	0.0	0.0	0.0	0.0
8	Sample #15 Dil. 4	0.0	0.0	0.0	0.0	0.0	0.0	0.0
9	Sample #15 Dil. 5	0.0	0.0	0.0	0.0	0.0	0.0	0.0
10	Sample #15 Dil. 6	0.0	0.0	0.0	0.0	0.0	0.0	0.0
11	Sample #15 Dil. 7	50.2	49.3	49.3	49.3	1.7	0.0	0.0
12	Sample #15 Dil. 8	0.0	0.0	0.0	0.0	0.0	0.0	0.0
13	Sample #15 Dil. 9	0.0	0.0	0.0	0.0	0.0	0.0	0.0
14	Sample #16 Dil. 1	0.0	0.0	0.0	0.0	0.0	0.0	0.0

15	Sample #16 Dil. 2	0.0	0.0	0.0	0.0	0.0	0.0	0.0
16	Sample #16 Dil. 3	0.0	0.0	0.0	0.0	0.0	0.0	0.0
17	Sample #16 Dil. 4	0.0	0.0	0.0	0.0	0.0	0.0	0.0
18	Sample #16 Dil. 5	0.0	0.0	0.0	0.0	0.0	0.0	0.0
19	Sample #16 Dil. 6	57.1	54.5	55.4	55.4	4.6	1.6	1.6
20	Sample #16 Dil. 7	21.7	20.5	20.5	20.5	5.4	0.0	0.0
21	Sample #16 Dil. 8	0.0	0.0	0.0	0.0	0.0	0.0	0.0
22	Sample #16 Dil. 9	0.0	0.0	0.0	0.0	0.0	0.0	0.0
23	Sample #17	14.3	10.2	20.5	20.5	40.4	101.6	101.6
24	Sample #18	0.0	0.0	0.0	0.0	0.0	0.0	0.0
25	Sample #19	0.0	0.0	0.0	0.0	0.0	0.0	0.0
26	Sample #20	0.0	0.0	0.0	0.0	0.0	0.0	0.0
27	Sample #21	0.0	0.0	0.0	0.0	0.0	0.0	0.0
28	Sample #23	0.0	0.0	0.0	0.0	0.0	0.0	0.0
29	Sample #24	0.0	0.0	0.0	0.0	0.0	0.0	0.0
30	Sample #34	0.0	0.0	0.0	0.0	0.0	0.0	0.0
31	Sample #35	0.0	0.0	0.0	0.0	0.0	0.0	0.0

32	Sample #36	0.0	0.0	0.0	0.0	0.0	0.0	0.0
33	Sample #37	0.0	0.0	0.0	0.0	0.0	0.0	0.0
34	Sample #38	0.0	0.0	0.0	0.0	0.0	0.0	0.0
35	Sample #39	0.0	0.0	0.0	0.0	0.0	0.0	0.0
36	Sample #40	0.0	0.0	0.0	0.0	0.0	0.0	0.0

$$\%\ \text{Difference} = 100 \times \frac{(\text{Paired or Random }\%\text{GCV-Singleton }\%\text{GCV})}{\text{Singleton }\%\text{GCV}}$$

%GCV, percent geometric coefficient of variation; HAI, hemagglutination inhibition; VLP, virus-like particle.

Figure S1: Correlation of Hemagglutination-Inhibition Titers Against A/Kansas/14/2017 in Clinical Trial Sera as Determined by Assays Using Wild-type VLPs and Egg-grown Viruses as Agglutinins.

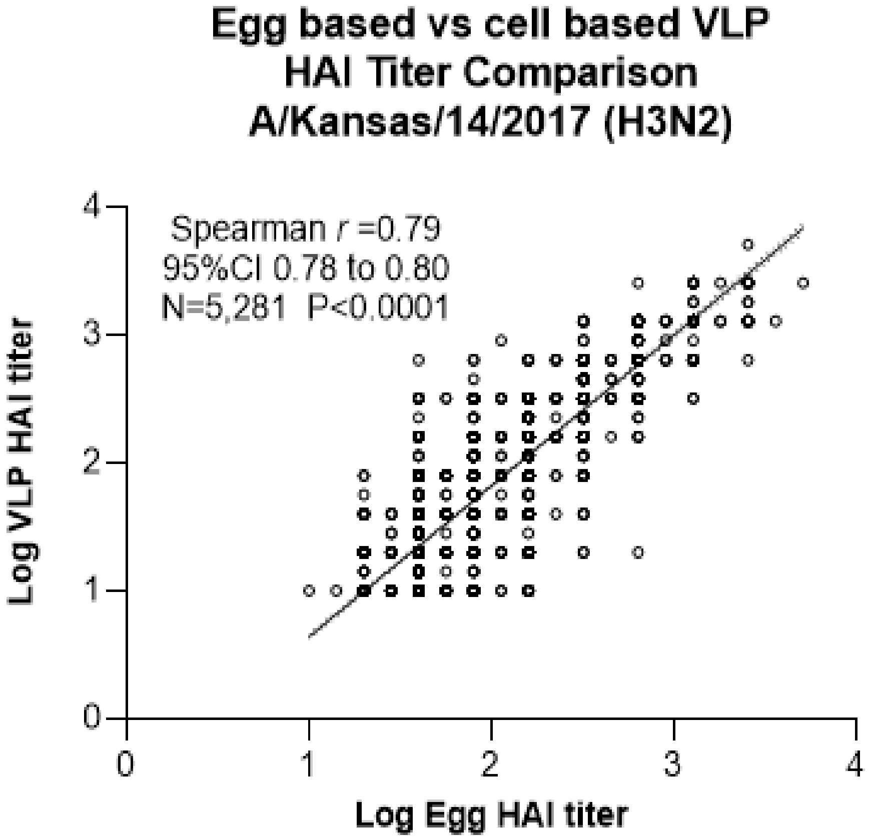

A total of 5,281 serum samples from 2,654 subjects in Novavax clinical trial qNIV-E-301 (Shinde, V., et al. 2022; doi:10.1016/S1473-3099(21)00192-4)), including approximately equal numbers of recipients of wild-type VLP and egg-grown inactivated virus 2019-20 Northern hemisphere vaccines, were tested in the novel HAI assay using egg-grown virus (*X*-axis) and cell-based (wild-type) VLP (*Y*-Axis) for A/Kansas/14/2017. Correlation data (Spearman *r* with 95% CI) analyzed using GraphPad PRISM (v10.2.0) is shown in the figure. The slope of the regression line was 1.18.

Table S14. Comparison of CypherOne™ Readout with the manual assay readout

Sample	Manual Read Titers	CypherOne Assay Titer	Results within 2-fold
Sample #1	10	10	Yes
Sample #2	40	40	Yes
Sample #3	20	20	Yes
Sample #4	10	10	Yes
Sample #5	80	80	Yes
Sample #6	40	40	Yes
Sample #7	160	160	Yes
Sample #8	20	20	Yes
Sample #9	5	5	Yes
Sample #10	320	320	Yes
Sample #11	80	80	Yes
Sample #12	80	80	Yes
Sample #13	80	80	Yes
Sample #14	40	40	Yes
Sample #15	40	40	Yes
Sample #16	320	320	Yes
**% of Samples within 2-fold**			***100%***
